# mCherry on Top: A Positive Read-Out Cellular Platform for Screening DMD Exon Skipping Xenopeptide–PMO Conjugates

**DOI:** 10.1021/acs.bioconjchem.3c00408

**Published:** 2023-11-22

**Authors:** Anna-Lina Lessl, Jana Pöhmerer, Yi Lin, Ulrich Wilk, Miriam Höhn, Elisa Hörterer, Ernst Wagner, Ulrich Lächelt

**Affiliations:** † Pharmaceutical Biotechnology, Department of Pharmacy, LMU Munich, Butenandtstrasse 5-13, 81377 Munich, Germany; ‡ Center for NanoScience (CeNS), LMU Munich, 80799 Munich, Germany; § Department of Pharmaceutical Sciences, University of Vienna, Josef-Holaubek-Platz 2, 1090 Vienna, Austria

## Abstract

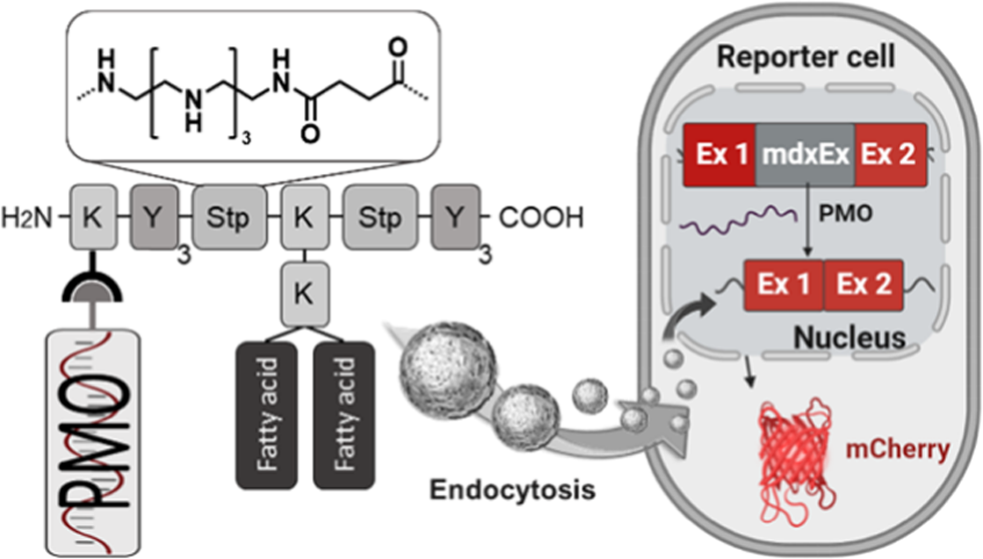

Phosphorodiamidate morpholino oligomers (PMOs) are a special type of antisense oligonucleotides (ASOs) that can be used as therapeutic modulators of pre-mRNA splicing. Application of nucleic-acid-based therapeutics generally requires suitable delivery systems to enable efficient transport to intended tissues and intracellular targets. To identify potent formulations of PMOs, we established a new *in vitro–in vivo* screening platform based on mdx exon 23 skipping. Here, a new *in vitro* positive read-out system (mCherry-DMDEx23) is presented that is sensitive toward the PMO(Ex23) sequence mediating DMD exon 23 skipping and, in this model, functional mCherry expression. After establishment of the reporter system in HeLa cells, a set of amphiphilic, ionizable xenopeptides (XPs) was screened in order to identify potent carriers for PMO delivery. The identified best-performing PMO formulation with high splice-switching activity at nanomolar concentrations *in vitro* was then translated to *in vivo* trials, where exon 23 skipping in different organs of healthy BALB/c mice was confirmed. The predesigned *in vitro*–*in vivo* workflow enables evaluation of PMO(Ex23) carriers without change of the PMO sequence and formulation composition. Furthermore, the identified PMO–XP conjugate formulation was found to induce highly potent exon skipping *in vitro* and redistributed PMO activity in different organs *in vivo*.

## Introduction

Therapeutic nucleic acids provide the opportunity for causal treatments of diseases by modifying the gene expression of disease-associated genes. One strategy for interference with the endogenous flow of genetic information is based on antisense oligonucleotides (ASOs), which bind to complementary mRNA sequences and induce gene knockdown or the modulation of pre-mRNA splicing. ASOs that modulate splicing are termed splice-switching oligonucleotides (SSOs) and can induce specific exclusion or inclusion of one or more exons as well as modulation of the equilibrium between splicing isoforms.^1−3^ A specific ASO chemistry is represented by phosphorodiamidate morpholino oligomers (PMOs) that are artificial nucleic acid analogues consisting of uncharged phosphorodiamidates and morpholine rings in the backbone. PMOs represent promising candidates for the treatment of several diseases such as viral infections,^4^ thalassemia,^5,6^ neuromuscular disorders,^7−11^ inflammation,^12^ retinopathies,^13^ and cancer.^14,15^ Eteplirsen, golodirsen, viltolarsen, and casimersen are approved PMO therapeutics used for the treatment of different genetic variants of Duchenne muscular dystrophy (DMD) *via* skipping of specific exon sequences.^16−23^ The synthetic uncharged character of PMOs has beneficial effects on stability, nuclease resistance, immunogenicity, and toxicity,^24,25^ but also influences the cellular uptake of naked PMO and compatibility with conventional nucleic-acid transfecting agents.^26^ To overcome these limitations, PMO conjugates with cell-penetrating peptides,^27−35^ dendrimers,^36,37^ cationic backbone modifications,^38^ and ionizable xenopeptides^39^ were established. The development of novel PMO formulations generally requires a set of complementary methods to assess splice switching on mRNA level, such as reverse transcription PCR (RT-PCR), as well as to confirm subsequent protein expression, for instance, via Western blotting. Furthermore, the *in vitro*–*in vivo* gap represents a critical hurdle for the translation of PMO formulations since physicochemical properties of PMOs can vary between different sequences. The Aoki lab has developed an EGFP reporter cell line and a related transgenic mouse model as an approach for the sensitive evaluation of mdx-type exon 23 skipping SSOs *in vitro* and *in vivo*.^40^ Here, we used an alternative *in vitro–in vivo* screening platform, based on DMD exon 23 skipping, which was utilized for the evaluation of xenopeptide (XP)–PMO conjugate formulations. To facilitate *in vitro* preselection, we designed a positive read-out system for detection of the splice-switching activity. An mCherry reporter gene was interrupted by the DMD exon 23 with a nonsense mutation, which is derived from the well-established murine DMD *mdx* model. Restoration of the reporter gene reading frame through *mdx* exon 23 skipping with the suitable PMO(Ex23) leads to functional mCherry expression that can be observed *via* fluorescence detection. In the current work, a library of XPs^41^ assembled from the artificial aminoethylene amino acid succinoyl tetraethylene pentamine (Stp), α-amino acids, and fatty acids was screened *in vitro* time-efficiently to identify highly potent PMO carriers. Since the reporter system was derived from the *mdx* model, screening results could directly lead to formulations applicable to transgenic *mdx* mice. Nevertheless, PMO(Ex23) also induces DMD exon 23 skipping in wild-type (wt) mice,^42^ and DMD is expressed in several major organs (brain, spleen, kidney, liver, lung, skeletal, and cardiac muscle). In consequence, the splice-switching activity of developed PMO conjugate formulations can also be evaluated in simple nontransgenic mouse models. Following this strategy, the work presents a convenient workflow for the development and *in vitro–in vivo* evaluation of PMO conjugate formulations. Furthermore, a potent XP conjugate formulation was identified, which features an altered *in vivo* biodistribution and tissue-specific activity of splice-switching PMOs.

## Results

### Screening Platform Design

**Figure 1:**
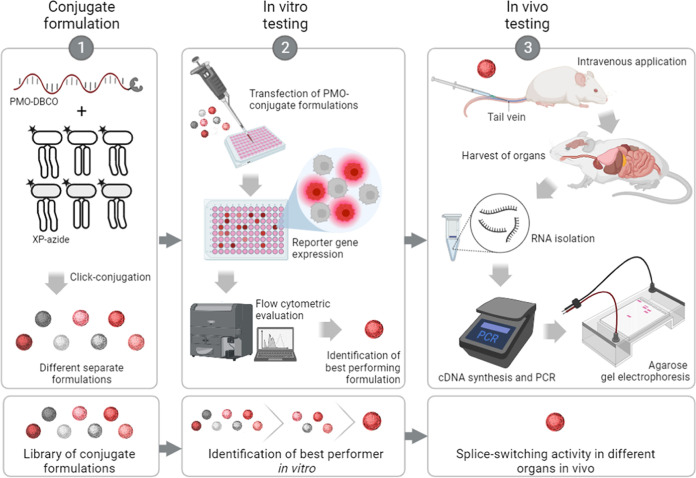
Schematic overview of the workflow from formulation generation to *in vitro* and *in vivo* evaluation. (1) Generation of PMO conjugate formulations using PMO(Ex23)-DBCO and azide-containing XPs *via* strain-promoted azide–alkyne cycloaddition (click conjugation). (2) *In vitro* screening of PMO conjugate formulations and identification of potent candidates. (3) Evaluation of favorable PMO conjugate formulations *in vivo* using the same PMO sequence and formulation composition.

**Figure 2:**
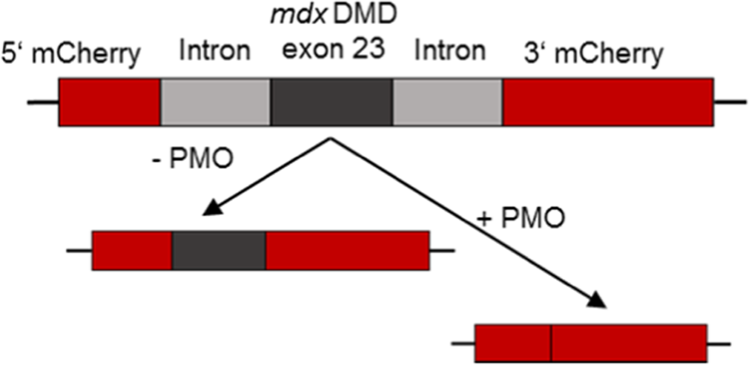
Structure of the mCherry-DMDEx23 construct and its mechanism in the presence or absence of PMO(Ex23).

During the development of nanotherapeutics, the translation from in *vitro* to *in vivo* stages is generally challenging. For PMO formulations, this gap can be particularly critical since different PMO sequences used in different screening models can exhibit very different physicochemical properties. For this reason, in this study, a workflow has been designed that enables *in vitro* and *in vivo* screening of PMO conjugate formulations without change of the sequence (Figure 1). The basis for the screening platform is the PMO(Ex23) sequence, which mediates skipping of DMD exon 23 and is frequently used in the well-established *mdx* mouse model of DMD. The particular PMO has the advantage that it can not only be used in transgenic *mdx* mice but does also induce exon skipping in wild-type mice. For initial assessments and preselections, an *in vitro* reporter system was designed, which enables convenient screening of PMO conjugate formulations. An intron–exon–intron sequence was implemented as the design of a fluorescent protein reporter in order to generate a positive read-out system activatable *via* exon skipping (Figure 2). Based on the *mdx* mouse model, the DMD exon 23 containing a nonsense mutation at nucleotides 28–30 was inserted as an interrupting exon into the *mCherry* gene between nucleotides 105 and 106. The exon is flanked by two artificial intronic sequences containing splice sites.

The donor splice site of the intron downstream of *mdx* exon 23 is based on the physiological donor splice site sequence of DMD intron 23, which enables utilization of the same PMO(Ex23) sequence in the *in vitro* reporter system and *in vivo* experiments. Transcription and splicing of the *mCherry-DMDEx23* construct results in mRNA with the nonsense mutation of *mdx* exon 23 and no functional mCherry production. Skipping of *mdx* exon 23 via a specific SSO such as PMO(Ex23) restores the correct reading frame and induces the functional mCherry expression. For the establishment of the reporter system, the functionality and suitability of the reporter gene for detecting DMD exon 23 skipping were first validated in HeLa cells transiently transfected with a pEGFP-N1/mCherry-DMDEx23 plasmid (Figure S1). For PMO conjugate formulation screenings, stable HeLa mCherry-DMDEx23 cells were generated with the PiggyBac system and a PB-mCherry-DMDEx23 plasmid (Figure S2) containing the reporter gene construct.

### mCherry-DMDEx23 Reporter System Validation

**Figure 3:**
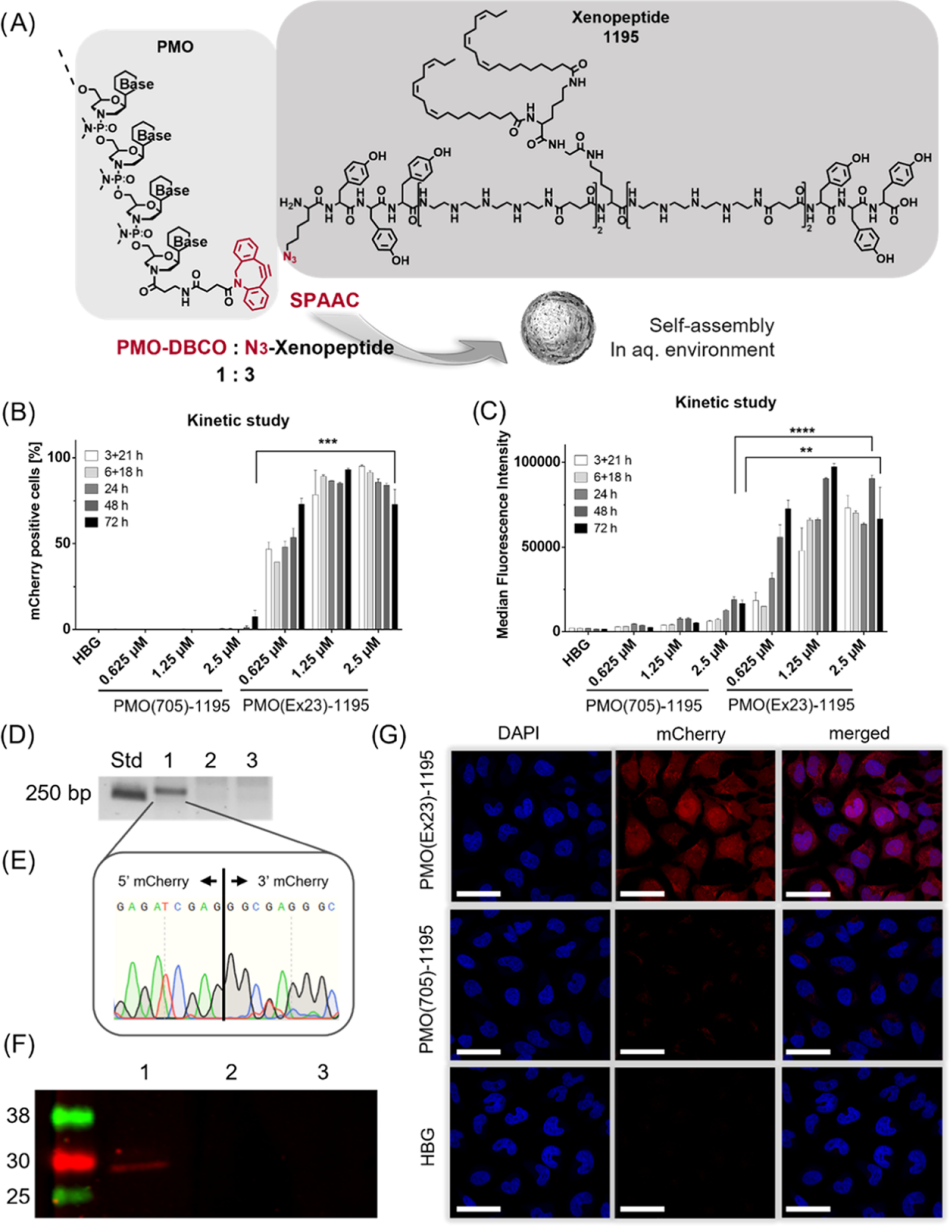
Validation of the reporter system. (A) Schematic illustration of strain-promoted azide–alkyne cycloaddition (SPAAC) conjugation of PMO–DBCO and azide-containing xenopeptide (XP) #1195 followed by nanomicelle formation. (B) mCherry-positive HeLa mCherry-DMDEx23 cells (%) after incubation with PMO(Ex23) or PMO(705) formulations between 3 (+21 h) and 72 h. Extended incubation times without PMO before evaluation are indicated in brackets. (C) Median mCherry fluorescence intensity (MFI) of HeLa mCherry-DMDEx23 cells after incubation with PMO(Ex23) or PMO(705) formulations between 3 (+21 h) and 72 h. Extended incubation times without PMO before evaluation are indicated in brackets. (D) Detection of mdx exon 23 skipping of mCherry-DMDEx23 mRNA by RT-PCR. Total RNA was extracted from cells 24 h after PMO(Ex23)-1195 and PMO(705)-1195 treatment (2.5 μM PMO). A sequence from mCherry-DMDEx23 after mdx exon 23 skipping was amplified by RT-PCR and the corresponding band is shown (∼280 bp). Std: DNA ladder; 1: PMO(Ex23)-1195; 2: PMO(705)-1195; 3: HBG. Complete gel is provided in Figure S6. (E) Exemplary Sanger sequencing results from the gel extracted band resulting from mdx exon 23 skipping. (F) Detection of the mCherry protein by Western blotting. HeLa mCherry-DMDEx23 cells were treated with PMO(Ex23)-1195 and PMO(705)-1195 formulations (2.5 μM PMO) for 24 h. (G) CLSM images of HeLa mCherry-DMDEx23 cells 24 h after transfection with PMO-1195 formulations containing PMO(Ex23)-DBCO or PMO(705)-DBCO, respectively (2.5 μM PMO). Nuclei were stained with DAPI (blue), and mCherry is shown in red. Scale bar is 50 μm. Statistical significance was estimated using a two-tailed student’s *t* test, ****p ≤ 0.0001, ***p ≤ 0.001, and **p ≤ 0.01. Data are presented as the mean ± SD (*n* = 3).

Kuhn et al. developed PMO conjugate formulations with lipid-modified xenopeptides (XPs) containing the ionizable artificial amino acid Stp, α-amino acids, fatty acids, and an azide function. The amphiphilic ionizable XP was assembled by solid-phase peptide synthesis (SPPS), including the integration of an azidolysine at the N-terminus. PMOs were functionalized with dibenzocyclooctyne (DBCO) *via* amidation at a 3′-primary amine to enable strain-promoted azide–alkyne cycloaddition (SPAAC)-driven conjugation of PMO–DBCO and the azide-containing XP (Figure 3A).^39,43,44^ It was shown that an excess of XPs in the XP–PMO conjugate formulations (molar ratio: 1:3 of PMO to XP) strongly enhances the transfection efficiency due to the formation of nanomicelles.^39^ Therefore, for the validation of the reporter system, PMO was used together with the previously reported XP 1195 (LP LenA) formulation in a 1:3 ratio. HeLa wt cells were transfected with the pEGFP-N1/mCherry-DMDEx23 plasmid, which contains the mCherry-DMDEx23 construct followed by an eGFP gene. Despite the location downstream of mCherry-DMDEx23, the eGFP expression enabled detection of transfected cells independent of mCherry, which could be explained by the additional Kozak consensus sequence and start codon between the reporter genes, a phenomenon described in the literature for fusion genes cloned into the pEGFP-N1 vector.^45^ The transiently transfected HeLa cells were subsequently treated with 1195 formulations of two different PMO sequences: PMO(Ex23) inducing DMD exon 23 skipping and as control PMO(705) without a complementary binding site. The extent of eGFP and mCherry expression was evaluated by flow cytometry (Figure S5). Direct comparison between the two PMO variants confirmed the sequence-specific response of the reporter system: treatment with the PMO(Ex23)-1195 1:3 formulation led to 20% mCherry-positive cells within the eGFP positive cell population, whereas treatment with PMO(705)-1195 1:3 did not induce mCherry expression. For convenient and reliable screenings of PMO formulations, a stable HeLa mCherry-DMDEx23 cell line was generated by utilizing the PiggyBac system and a PB-mCherry-DMDEx23 plasmid (Figure S2). The monoclonal reporter cell line was treated with PMO-1195 1:3 formulations at concentrations 2.5, 1.25, and 0.625 μM for 3 h (followed by 21 h incubation in fresh medium), 6 h (followed by 18 h incubation in fresh medium), 24, 48, and 72 h. The relative number of mCherry-expressing cells (Figure 3B) and the median mCherry fluorescence intensity (MFI) were determined by using flow cytometry (Figure 3C). The evaluation of mCherry expression showed a concentration and time-dependent effect, where the increase of concentration and incubation time led to a higher ratio of mCherry-positive cells, when treated with PMO(Ex23) formulations. In direct comparison, treatment with the control formulation PMO(705)-1195 1:3 did not lead to substantial mCherry expression. To confirm the generation of the supposed mRNA splicing product, a specific amplification approach was chosen: RNA was isolated from treated cells and reverse-transcribed into cDNA. A nested PCR was carried out with an Nde I restriction digestion step between the two amplifications. DMD exon 23 contains the recognition site of Nde I, which leads to preferential amplification of the DNA with skipped exon 23. This approach was chosen to selectively enrich the splicing product among others, which is responsible for functional mCherry expression. Gel electrophoresis was used to analyze the DNA samples obtained from HeLa mCherry-DMDEx23 cells treated with 2.5 μM PMO(Ex23)-1195 and PMO(705)-1195 1:3 or HBG buffer, respectively. A DNA band of the correct size (280 bp) was detected only in the case of PMO(Ex23)-1195-treated cells (Figure 3D) and Sanger sequencing confirmed the correct sequence of the mCherry reporter fragment after exon 23 skipping (Figure 3E). The presence of the mCherry protein after PMO(Ex23) delivery was further determined by Western blotting (Figure 3F) and confocal laser scanning microscopy (CLSM, Figure 3G). In both cases, only after treatment with the PMO-1195 formulation containing PMO(Ex23), mCherry protein was detectable.

### *In Vitro* Screening of PMO(Ex23) Formulations with Xenopeptides

**Figure 4:**
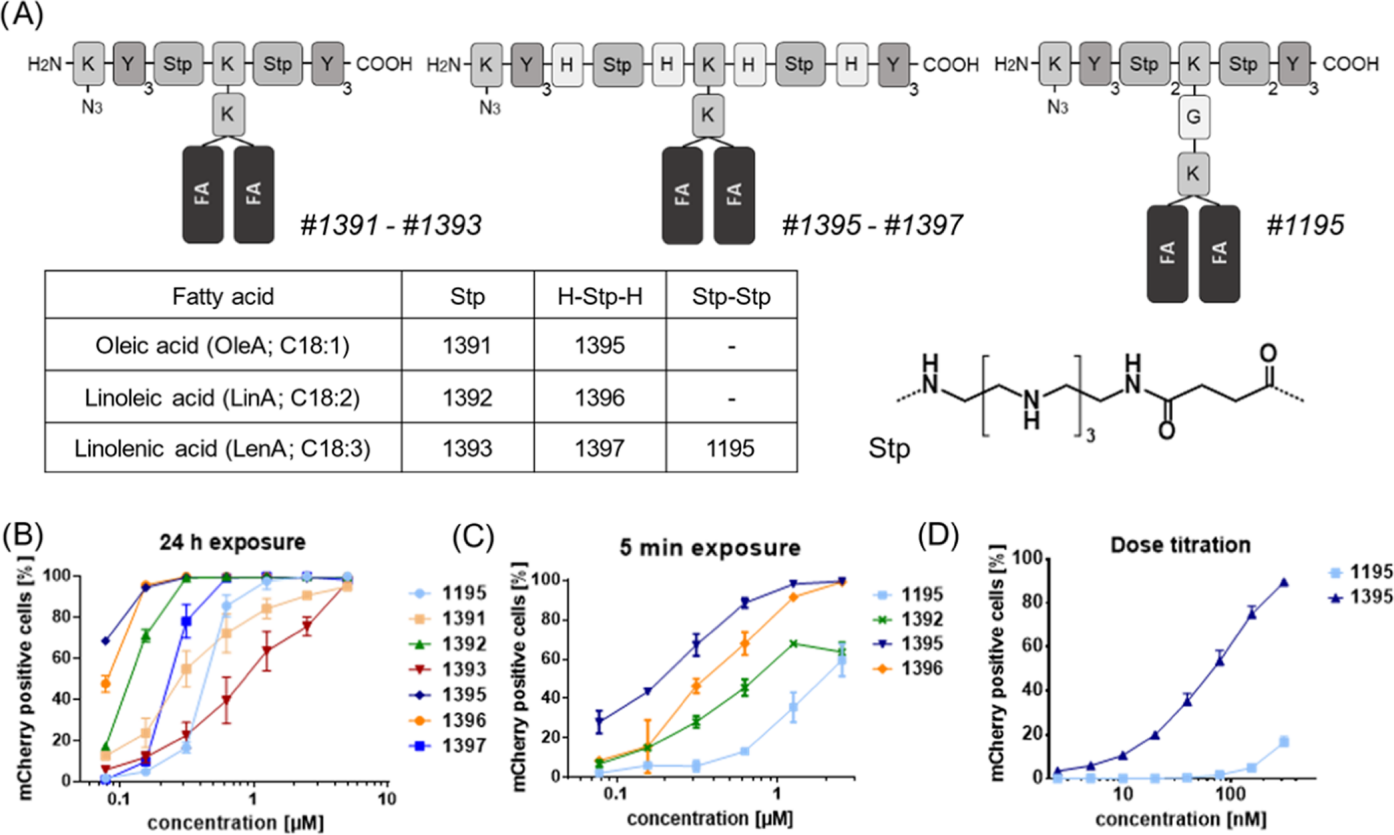
Structure–activity relationship of PMO(Ex23)–XP 1:3 formulations. (A) Schematic illustrations of XP architectures classified according to the ionizable backbone into “Stp” (#1391-#1393), “H-Stp-H” (#1395-#1397), and “Stp-Stp” (#1195). The table summarizes individual fatty acids contained. K: lysine, Y: tyrosine; H, histidine; Stp: succinoyl tetraethylene pentamine; FA: fatty acid. (B) Splice-switching activity in HeLa mCherry-DMDEx23 cells after 24 h treatment with PMO(Ex23)–XP 1:3 formulations (0.078–5 μM PMO). (C) Splice-switching activity in HeLa mCherry-DMDEx23 cells after 5 min exposure to PMO(Ex23)–XP 1:3 formulations (0.078–2.5 μM PMO) and subsequent incubation in fresh medium for 24 h. Additional exposure times (15 and 30 min) are provided in Figure S8. (D) Dose titration of PMO(Ex23)-1195 and -1395 formulations (2.4–312.5 nM PMO) with exposure of HeLa mCherry-DMDEx23 cells for 24 h. Percentage of mCherry-expressing cells was determined 24 h after treatment. Data are presented as the mean ± SD (*n* = 3).

To identify new potent carriers for PMO delivery, a small library of XPs with structural variations was screened (Figure 4A). PMO(Ex23)–DBCO conjugate formulations with each oligomer were prepared in a molar ratio of 1:3 (PMO/XP). In direct comparison to the previous lead structure #1195, five alternative XPs exhibited much higher potency. Histidine-containing XPs generally mediated higher transfection efficiencies even at low concentrations. Oleic acid and linoleic acid (#1395 and #1396) were found to be favorable structural elements in the new XP architectures (Figure 4B).

**Figure 5:**
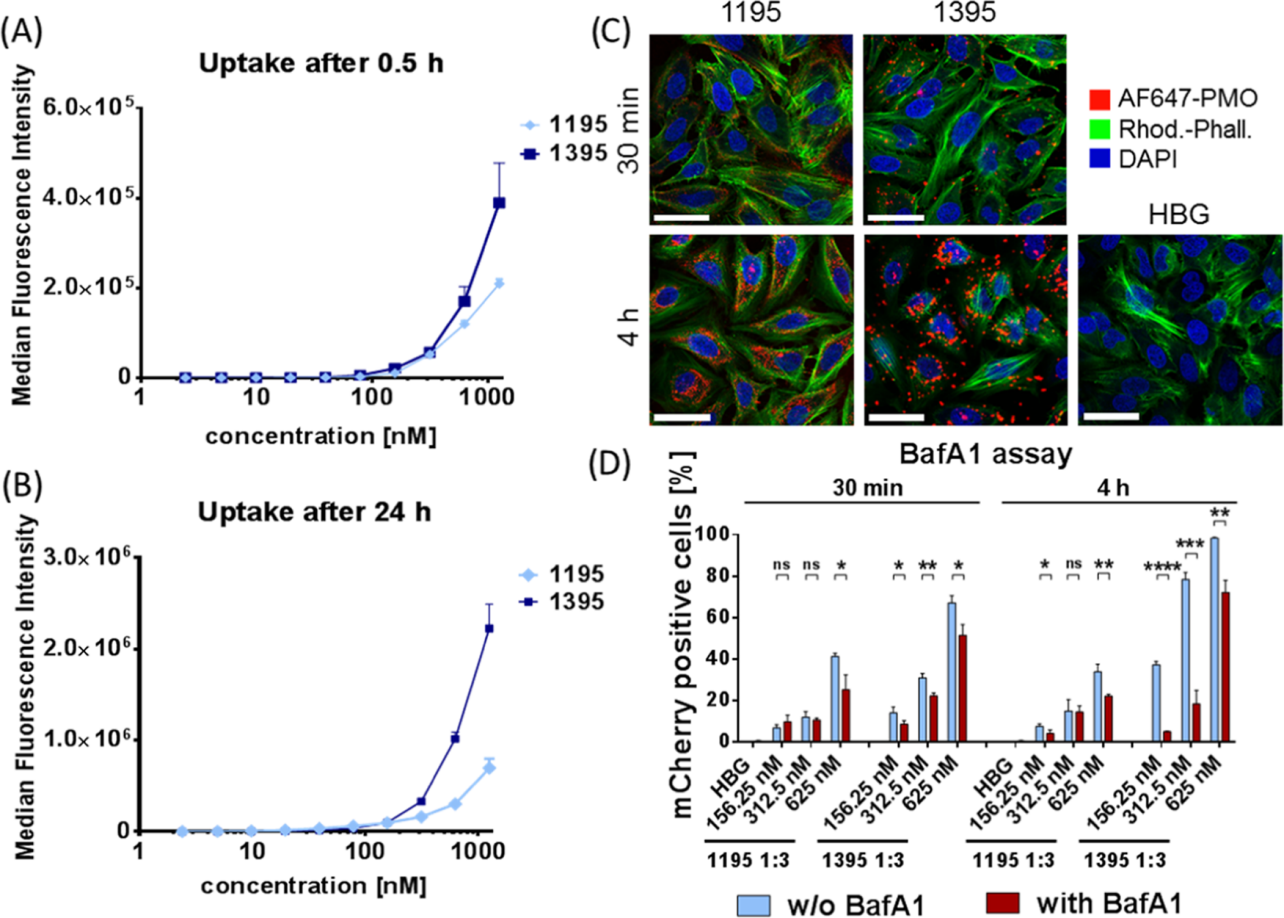
Cellular uptake of PMO–XP conjugate formulations. (A, B) Cellular uptake of AF647-labeled PMO in HeLa mCherry-DMDEx23 cells 0.5 h (A) and 24 h (B) after treatment with PMO-1195 or PMO-1395 formulations containing 5% AF647-PMO(705) and 95% PMO(Ex23). Total PMO concentrations ranged from 2.4 to 1250 nM. (C) CLSM images of HeLa wt cells after treatment with PMO-1195 and PMO-1395 formulations (5% AF647-PMO(705), 95% PMO(Ex23); 2.5 μM total PMO). Nuclei were stained with DAPI (blue) and actin filaments with rhodamine phalloidin (green). AF647 fluorescence is indicated in red. Scale bar represents 50 μm. (D) Influence of endosomal acidification on the splice-switching activity of PMO–XP conjugate formulations determined by V-ATPase inhibition with bafilomycin A1 (BafA1). HeLa mCherry-DMDEx23 cells were preincubated with BafA1 (200 nM) for 2 h and cells were treated with PMO(Ex23)-1195 or -1395 formulations. Medium was replaced by fresh medium 30 min or 4 h after treatment and mCherry-positive cells were quantified after a total incubation time of 24 h. Median fluorescence intensity (MFI) data of mCherry-positive cell populations are provided in Figure S10. Statistical significance was estimated using the two-tailed student’s *t* test, ****p ≤ 0.0001, ***p ≤ 0.001, **p ≤ 0.01, and *p ≤ 0.05. ns *p* > 0.05. Data are presented as the mean ± SD (*n* = 3).

Over 90% of cells were mCherry-positive after treatment with the most potent #1395 and #1396 formulations containing 156.25 nM PMO (Figure 5B). In all cases, the ratio of mCherry-expressing cells increased with an increasing PMO concentration. At the same time, a dose-dependent cytotoxicity at higher concentration was observed (Figure S7) which illustrates the need for potent formulations with high activity at low doses. The best-performing PMO formulations were based on XP #1392, #1395, and #1396 and were additionally compared to the previously established #1195 formulation in experiments with short exposure times of 5 min (Figure 4C), 15 min (Figure S8A), and 30 min (Figure S8B). After the indicated exposure to PMO formulations, cells were incubated in fresh medium until 24 h since starting the experiment. Especially, the XP #1395 and #1396 formulations turned out to be very potent and resulted in >45% mCherry-positive cells even after exposure to a moderate PMO dose of 312.5 nM for 5 min only. Transmission electron microscopy (TEM) illustrated the self-association of PMO(Ex23)-1395 1:3 formulations into nanoparticles, similar to the previously published XP 1195 (Figure S4).

To assess the favorable properties of PMO(Ex23)-1395 formulations in more detail, an additional comparison with PMO(Ex23)-1195 was carried out at low PMO concentrations between 2.4 and 312.5 nM. HeLa mCherry-DMDEx23 cells were treated for 24 h (Figure 4D). The dose–response curves clearly illustrate the much higher potency of #1395, which mediates splice switching already at PMO concentrations ≤5 nM. The screening result obtained from the HeLa mCherry-DMDEx23 system was also validated in an alternative commonly used splice-switching reporter model. HeLa pLuc/705 cells are based on the pLuc/705 construct developed by Ryszard Kole’s lab in the 1990s, which indicates successful skipping of mutated β-globin intron IVS2-705 via luciferase expression.^46^ With PMO(705)–XP formulations, targeting the mutated site of the pLuc/705 transcript, tendencies similar to those in HeLa mCherry-DMDEx23 cells were found, and XP #1395 was confirmed as the best-performing PMO formulation (Figure S9).

### Cellular Uptake of 1395 Formulations

The high splice-switching activity of the #1395 formulation suggests a favorable cellular uptake of the contained PMO cargo. To assess the difference of #1195 and #1395, cellular uptake of formulations containing 5% Alexa Fluor 647 (AF647)-labeled PMO into HeLa mCherry-DMDEx23 cells was determined by flow cytometry after 0.5 and 24 h incubation time (Figure 5A,B). Both formulations showed time- and concentration-dependent uptake characteristics, and at both time points, the direct comparison confirmed a higher extent of PMO internalization mediated by the #1395 formulation, which correlates with the increased splice-switching activity. The observation that effects of PMO(Ex23) treatment on splicing modulation could already be detected at much lower concentrations in HeLa mCherry-DMDEx23 cells can be explained by two reasons: first, the detected AF647 signal in the case of uptake experiments results from 5% AF647-labeled PMO in the formulation, whereas each PMO(Ex23) molecule can be active in splice-switching experiments. Second, PMO(Ex23) causes an amplifying effect on mCherry expression, since each molecule can modulate splicing of several mCherry-DMDEx23 pre-mRNAs and each mature mRNA can be translated into protein repeatedly. In contrast, the intensity of AF647 is directly correlated with the PMO concentration. These considerations translate into the high sensitivity of the mCherry-DMDEx23 reporter system. Additionally, confocal laser scanning microscopy (CLSM) experiments confirmed the beneficial cellular uptake of PMO-1395 conjugate formulations (Figure 5C). After endocytotic uptake, nanoparticles are frequently entrapped in endosomes, which hampers the reach of other intracellular compartments. The internal volume of endosomes represents an acidic environment due to the activity of proton pumps, which is a frequently used trigger for inducing endosomolytic or fusogenic properties of delivery systems. To elucidate the impact of low endosomal pH on the delivery efficiency, treatments of HeLa mCherry-DMDEx23 cells with PMO(Ex23)-1195 and PMO(Ex23)-1395 conjugate formulations were performed in the presence and absence of bafilomycin A1 (BafA1). BafA1 is an inhibitor of vacuolar-type H^+^-ATPase (V-ATPase) and reduces endosomal acidification. Previous works could show that transfection with delivery systems based on ionizable structural motifs can be inhibited by BafA1.^47−52^ By using the mCherry-DMDEx23 reporter cells, we evaluated the dependency of successful PMO delivery on the endosomal pH on a functional level.

The transfection efficiencies of #1195 as well as #1395 formulations were decreased in the presence of BafA1 compared to the controls without BafA1 (Figure 5D). Especially, after 4 h PMO exposure time, BafA1 had significant influence on the #1395 formulations. These findings led to the conclusion that the evaluated PMO–XP conjugate formulations depend on the endosomal acidification during the intracellular delivery process.

### *In Vivo* Evaluation

**Figure 6:**
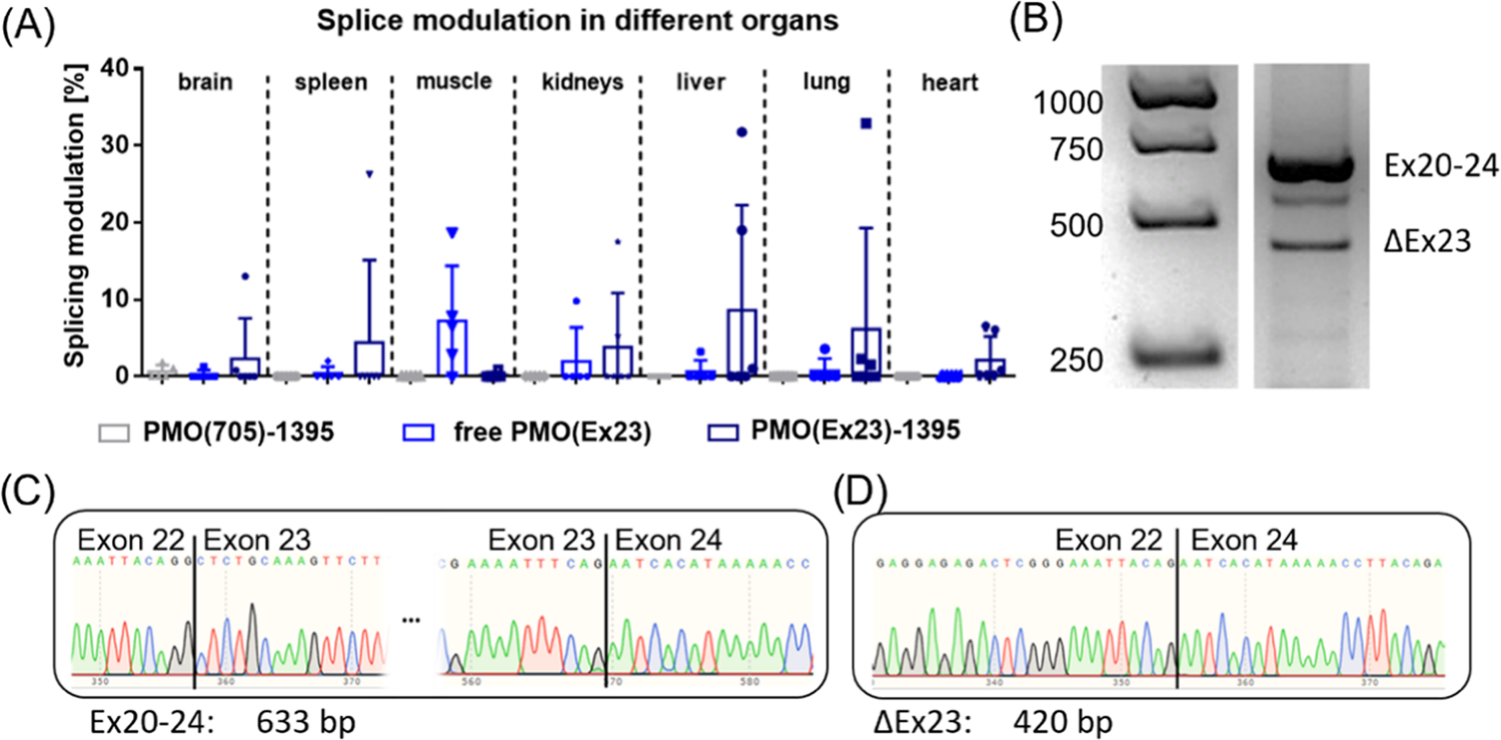
*In vivo* mRNA splicing modulation in BALB/c mice. (A) Comparison of DMD mRNA splicing modulation in different organs of BALB/C mice determined *ex vivo* by RT-PCR 48 h after intravenous injection of PMO(Ex23)-1395, PMO(705)-1395 formulations, or unformulated PMO(Ex23) at a PMO dose of 375 μg. Total RNA was extracted from homogenized organs and nested RT-PCR was conducted to amplify DMD Ex20–24. (PMO(705)-1395, *n* = 5; free PMO(Ex23)-DBCO, *n* = 5; PMO(Ex23)-1395, *n* = 6). The complete gel electrophoresis and individual splicing modulation data are provided in Figure S11. (B) Exemplary gel electrophoresis result showing splicing modulation in the lung of animal C1 treated with PMO(Ex23)-1395. (C, D) Sanger sequencing of gel extracted bands corresponding to the 633 bp (C) and 420 bp (D) fragments. Expected sequences of complete DMD Ex20–24 (633 bp, C) and after DMD exon 23 skipping (420 bp, D) were found.

The *in vitro* screening identified the highest PMO delivery potency for the PMO-1395 conjugate formulation, which was therefore selected for *in vivo* evaluation. PMO(Ex23)-1395 as well as PMO(705)-1395 and unformulated PMO(Ex23) were intravenously injected into BALB/c mice. After 48 h, DMD exon 23 skipping in brain, spleen, kidney, liver, lung, heart, and quadriceps femoris muscles was evaluated by RT-PCR *ex vivo*. While unformulated PMO(Ex23) showed predominant splice-switching activity in the skeletal muscle, #1395 led to a redistribution to different organs: with the PMO(Ex23)-1395 formulation exon skipping in the skeletal muscle was reduced; instead activity was observed in spleen, kidneys, liver, lung, and some splicing modulation was even observed in brain and heart (Figure 6A). Representative Sanger sequencing of the RT-PCR products isolated from an agarose gel (633 and 420 bp bands of animal C1, lung; Figure 6B) confirmed the desired exon 23 skipping in the physiological dystrophin mRNA (Figure 6C, 633 bp band; Figure 6D, 420 bp band). However, it has to be noted that the PMO(Ex23)-1395 group exhibited high variability and individual animals showed high splicing modulation, in contrast to others. It is speculated that the high standard deviation could be caused by a suboptimal reconstitution of freeze-dried PMO formulations at high concentrations for *in vivo* application. In this case, further optimization of the formulation of concentrated *in vivo* samples, for instance, by using cryo- and lyoprotectants during the freeze-drying process could address issues with nanoformulation homogeneity in the future.

## Conclusions

In this study, we developed a new positive read-out cellular system based on mCherry and the murine *mdx* model to facilitate SSO formulation screenings. The sequence specificity, sensitivity, and fast kinetics of mCherry expression were demonstrated, which makes the mCherry-DMDEx23 a suitable reporter system for convenient SSO screenings. The application of the mCherry-DMDEx23 reporter was demonstrated by the screening of a series of XPs for PMO delivery. Potent XP–PMO formulations could be identified containing a more hydrophobic, ionizable backbone, which mediates splice switching at low nanomolar concentrations. Following a consequent *in vitro–in vivo* workflow, the best-performing formulation PMO(Ex23)-1395, identified *in vitro*, was subsequently tested *in vivo*. By using RT-PCR of *ex vivo-*isolated mRNA, skipping of DMD exon 23 was confirmed in the spleen, kidneys, liver, and lung as well as to a minor extent in brain and heart of BALB/c mice. In sum, the work presents a convenient workflow for the development of SSO formulations based on a combination of the new DMD exon 23 skipping reporter and physiological DMD exon 23 skipping in mice. Furthermore, highly potent PMO formulations are reported, which cause a redistribution of PMO *in vivo* and could enable splicing modulation in tissues beyond skeletal muscle.

## Experimental Procedure

### Generation of PMO–Xenopeptide Conjugate Formulations

For the conjugation of PMO–DBCO and xenopeptides (XP) at a molar ratio of 1:3 PMO to XP, a dilution of PMO–DBCO with a concentration of 100 μM in water and a dilution of the XP with a concentration of 300 μM in water were prepared. Equal volumes of the solutions were combined and incubated overnight at room temperature (RT) with shaking at 300 rpm. The formulation solution was freeze-dried and reconstituted with the required volume of HBG to obtain the desired concentration. For fluorescently labeled PMO–XP conjugate formulations, 5% of PMO(Ex23)–DBCO was replaced by Alexa Fluor 647 (AF647)-labeled PMO(705)–DBCO.^39^

### Generation of mCherry Reporter for DMD Exon 23 Skipping

The mCherry sequence was split into two parts: 5′-mCherry and 3′-mCherry. The two regions were separated by the murine exon 23 sequence containing the nonsense mutation of the *mdx* mouse model, flanked by intronic sequences of 154 bp at each side. The sequence of the reporter construct is provided in the SI (Figure S3). The construct was synthesized and cloned into a pEGFP-N1 vector by BioCat (Heidelberg, Germany). The pEGFP-N1/mCherry-DMDEx23 plasmid was used for initial transient reporter expression experiments; additionally, the gene construct was subcloned into a modified PiggyBac plasmid (PB-CAG-GFPd2 was a gift from Jordan Green; Addgene plasmid #115665; http://n2t.net/addgene:115665; RRID: Addgene_115665),^53^ which was used for generation of HeLa cells with stable reporter expression. The plasmid maps and description of stable cell line generation are provided in the SI. Splicing reporter plasmids pEGFP-N1/mCherry-DMDEx23 (#211367) and PB-mCherry-DMDEx23-eGFP (#211366) are deposited at Addgene.

### Cell Culture

HeLa wt and HeLa mCherry-DMDEx23 cells were cultured in DMEM (Sigma-Aldrich, St. Louis) supplemented with 10% fetal bovine serum (FBS; Life Technologies, Carlsbad) and 1% penicillin/streptomycin (P/S; Life Technologies, Carlsbad) at 37 °C and 5% CO_2_ in a humidified atmosphere. The medium was changed every 2 days, and cells were passaged at a confluency of approximately 80%.

### Flow Cytometry

Flow cytometry was used for the determination of mCherry expression as well as the cellular uptake of fluorescently labeled PMO formulations in HeLa mCherry-DMDEx23 cells. After individual treatments, cells were trypsinized and resuspended in 100 μL of FACS buffer, consisting of 10% FBS in PBS supplemented with DAPI (1 μg/mL) to discriminate viable and dead cells. All samples were analyzed by flow cytometry using a CytoFLEX S flow cytometer (Beckman Coulter, Carlsbad) equipped with a well-plate autosampler. DAPI was excited at 405 nm, and emission was detected at 450 nm. In splice-switching experiments, mCherry was excited at 561 nm, and emission was detected at 610 nm. In the case of uptake experiments, AF647-labeled PMO was excited at 640 nm and emission was detected at 670 nm. Only isolated and viable cells were evaluated. Flow cytometry data were analyzed using FlowJo X 10.0.7r2 flow cytometric analysis software by FlowJo, LLC (Ashland). All experiments were performed in triplicate.

### *In Vitro* Treatment of Transient HeLa mCherry-DMDEx23 Cells

Hela wt cells were seeded into a 96-well plate 24 h prior to transfection at a density of 5000 cells/well. 200 ng of pEGFP-N1/mCherry-DMDEx23 plasmid was transfected into each well using Lipofectamine 3000 (Thermo Fisher Scientific, Waltham) according to the manufacturer’s protocol. 24 h after the plasmid DNA transfection, cells were treated with the specified PMO formulations. The medium of the cells was replaced by 90 μL of fresh medium, and 10 μL of the PMO formulation was added. After 24 h of incubation, the medium was removed, each well was washed with 100 μL of PBS, and the cells were analyzed by flow cytometry using a CytoFLEX S flow cytometer (Beckman Coulter, Carlsbad) as described above.

### *In Vitro* Treatment of Stable HeLa mCherry-DMDEx23 Cells

Stable HeLa mCherry-DMDEx23 cells were seeded into 96-well plates (Corning Costar, Sigma-Aldrich, Munich, Germany) 24 h prior to treatments at a density of 5000 cells/well. The medium in each well was replaced by 90 μL of fresh medium, 10 μL of the PMO–XP conjugate formulation was added into each well, and the cells were exposed to the formulation for the specified incubation times. For short-term incubations, the medium containing the PMO–XP conjugate formulation was replaced by fresh medium after the desired incubation time, and incubation was continued for a total time of 24 h. After 24 h incubation, the medium was removed, each well was washed with 100 μL of PBS, and cells were analyzed by flow cytometry using a CytoFLEX S flow cytometer (Beckman Coulter, Carlsbad) as described above.

### Cellular Uptake Determined by Flow Cytometry

Stable HeLa mCherry-DMDEx23 cells were seeded into 96-well plates (Corning Costar, Sigma-Aldrich, Munich, Germany) 24 h prior to treatments at a density of 5000 cells/well. The medium in each well was replaced by 90 μL of fresh medium, 10 μL of the PMO–XP conjugate formulation containing 5% AF647-labeled PMO was added into each well, and the cells were incubated for 0.5 or 24 h, respectively. After the specified time, the medium was removed, each well was washed with 100 μL of PBS, and the cells were analyzed by flow cytometry using a CytoFLEX S flow cytometer (Beckman Coulter, Carlsbad) as described above.

### Metabolic Activity Assay

Cell viability was determined indirectly via the quantification of cellular metabolic activity with MTT assays. HeLa mCherry-DMDEx23 cells were seeded in 96-well plates at a density of 5000 cells/well. 24 h after seeding, the medium was replaced by 90 μL of fresh medium, and 10 μL of the PMO–XP conjugate formulation at the desired concentration was added to each well. After incubation for 24 h, 10 μL of 3-(4,5-dimethylthiazol-2-yl)-2,5-diphenyltetrazolium bromide (MTT, 5 mg/mL) was added to each well. After 2 h of incubation, the medium was removed, and the 96-well plates were stored at −80 °C overnight. 100 μL of DMSO was added per well to dissolve the purple formazan product. The 96-well plates were incubated for 30 min at 37 °C with constant shaking. The absorbance at λ = 590 nm of each well was measured with background correction at λ = 630 nm by using a microplate reader (Tecan Spark 10M, Tecan, Nänikon, Switzerland). The relative cell viability (%) was calculated by normalizing the values to control wells treated with HBG, according to the following equation
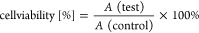
Data are reported as means ± standard deviation.

### mCherry Expression Imaged by CLSM

15 000 HeLa mCherry-DMDEx23 cells/well were seeded in 8 well-Ibidi μ-slides (Ibidi, Planegg/Martinsried, Germany) in a total volume of 300 μL medium per well. The next day, the medium was replaced by 270 μL of fresh medium. PMO–XP conjugate formulations in HBG were prepared at 25 μM as explained above, and 30 μL was added to each well. 24 h after the treatment, the wells were washed three times with 300 μL of prewarmed PBS and cells were fixed with 4% paraformaldehyde in PBS for 40 min at RT. The wells were washed again three times with PBS. The cell nuclei were stained with DAPI (2 μg/mL) for 20 min under light protection at RT. The wells were washed once with PBS and 300 μL of fresh PBS was added. A Leica-TCS-SP8 confocal laser scanning microscope (CLSM) equipped with an HC PL APO 63× 1.4 objective (Wetzlar, Germany) was used to record the images. DAPI emission was recorded at 460 nm, and mCherry emission was recorded at 610 nm. All images were processed by using the LAS X software from Leica.

### Uptake of PMO–XP Conjugate Formulations Imaged by CLSM

15 000 HeLa mCherry-DMDEx23 cells/well were seeded in 8 well-Ibidi μ-slides (Ibidi, Planegg/Martinsried, Germany) in a total volume of 300 μL medium per well. Cells were incubated at 37 °C and 5% CO_2_ for 24 h before treatment with PMO–XP conjugate formulations containing 5% AF647-labeled PMO. The next day, the medium was replaced by 270 μL of fresh medium and 30 μL of a 25 μM PMO–XP conjugate formulation solution in HBG was added per well. 0.5 or 4 h after the treatment, the wells were washed thrice with 300 μL of prewarmed PBS and cells were fixed with 4% paraformaldehyde in PBS for 40 min at RT. The wells were washed again thrice with PBS. F-actin was stained with rhodamine phalloidin (1 μg/mL) overnight at 4 °C. Cell nuclei were stained with DAPI (2 μg/mL) for 20 min under light protection at RT. The wells were washed once with PBS and 300 μL of fresh PBS was added. A Leica-TCS-SP8 confocal laser scanning microscope (CLSM) equipped with an HC PL APO 63× 1.4 objective (Wetzlar, Germany) was used to record the images. DAPI emission was recorded at 460 nm, rhodamine at 580 nm, and AF647 at 665 nm. All images were processed using the LAS X software from Leica.

### Western Blot

150 000 HeLa mCherry-DMDEx23 cells/well were seeded into a 6-well plate 24 h prior to PMO treatments. PMO formulations containing PMO(Ex23)-DBCO or PMO(705)-DBCO at a concentration of 25 μM were prepared with XP #1195 as described above. Prior to the treatment, the medium was replaced by 900 μL of fresh medium and 100 μL of PMO formulations were added into the wells. After an incubation time of 24 h, cells were washed with PBS and lysed using 100 μL of 0.5× cell culture lysis buffer (Promega, Mannheim, Germany) supplemented with EDTA-free Protease Inhibitor Cocktail (Roche, Basel, Switzerland) per well. The cell lysates were stored at −80 °C for 18 h. After centrifugation for 10 min at 4 °C and 17 000*g*, 40 μL of a 1:4 dilution of each supernatant was added to the SDS sample buffer containing β-mercaptoethanol and incubated for 5 min at 95 °C and 2 min on ice. SDS-PAGE was performed using a 3.5% stacking and a 10% separating SDS-gel. The proteins were transferred to a poly(vinylidene difluoride) (PVDF) blotting membrane. The membrane was incubated in 1× NET-gelatin (8.33 g of gelatina alba in 1 L of 1× NET containing 0.15 M NaCl, 0.005 M EDTA, 0.05 M Tris, and 0.05% Triton-X-100) for 2 h at room temperature and immunolabeled with a primary anti-mCherry antibody (anti-mCherry Rabbit polyclonal antibody, #26765-1-AP, Proteintech, Manchester, UK) diluted 1:1000 in 1× NET-gelatin overnight at 4 °C. The membrane was washed four times with 1× NET-gelatin and incubated with the secondary antibody (IRDye 680RD Goat antirabbit, Li-Cor, Lincoln) diluted 1:10 000 in 1× NET-gelatin for 1 h at RT followed by four times washing with 1× NET-gelatin. Membranes were imaged using an Odyssey Fa imaging system (Li-Cor, Lincoln) and analyzed and quantified by Image Studio Software (Li-Cor, Lincoln).

### Bafilomycin A1 Assay

5000 HeLa mCherry-DMDEx23 cells/well were seeded in 96-well plates 1 day prior to treatments. Two hours before PMO treatment, the medium in the wells was replaced by either fresh medium or medium supplemented with bafilomycin A1 (BafA1, Sigma-Aldrich, Munich, Germany) to obtain a final concentration of 200 nM BafA1 after PMO formulation addition. After 2 h of preincubation with BafA1, the PMO formulations at concentrations 6.25, 3.125, and 1.5625 μM were added as explained above. Cells were incubated for 30 min and 4 h, respectively, and the medium was replaced by 100 μL of fresh medium. The cells were incubated for further 23.5 or 20 h, respectively, before determination of mCherry expression by flow cytometry as described above. Treatments were performed in triplicate.

### RT-PCR of Reporter Exon Skipping *In Vitro*

Total RNA was isolated from PMO-treated and untreated HeLa mCherry-DMDEx23 cells using RNASolv (VWR International, Darmstadt, Germany) according to the manufacturer’s protocol. 400 ng of RNA was used to generate cDNA using the qScript cDNA synthesis kit (Quantabio, Beverly) according to the manufacturer’s protocol. The region of interest was amplified by PCR with 150 ng of the cDNA, Taq polymerase (New England Biolabs, Ipswich), the primers mCherry-DMDex23_SpliSwi_fwd (5′-GGAGGATAACATGGCCATCA-3′) and mCherry-DMDex23_SpliSwi_rev (5′-GTCCTTCAGCTTCAGCCTCT-3′), and the following PCR conditions: initial denaturation (94 °C, 30 s), 30 cycles (94 °C, 30 s/60 °C, 1 min/68 °C, 1 min), and final extension (68 °C, 5 min).

After amplification, the PCR product was purified using a PCR purification kit (Qiagen, Hilden, Germany) according to the manufacturer’s protocol. 1 μg of the purified PCR amplicon was digested overnight with 2 units of Nde I (New England Biolabs, Ipswich). To ensure detachment of the restriction enzyme, SDS was added to a concentration of 0.2% and was incubated for 10 min at 65 °C. The digested DNA was purified using the PCR purification kit (Qiagen, Hilden, Germany). Second PCR was conducted with 100 ng of the purified DNA as template, the primers mCherry-DMDex23_SpliSwi_nested_fwd (5′-GGAGTTCATGCGCTTCAAGG-3′) and mCherry-DMDex23_SpliSwi_nested_rev (5′-GCCGTCCTCGAAGTTCATCA-3′), and the following PCR conditions: initial denaturation (94 °C, 30 s), 30 cycles (94 °C, 30 s/55 °C, 1 min/68 °C, 1 min), and final extension (68 °C, 5 min).

The PCR product was analyzed on a 1% agarose gel in 1× TBE buffer containing GelRed (Biotium, Hayward). Electrophoresis was conducted for 1.5 h at 100 V and analyzed with Dark Hood DH-40 (biostep, Burkhardtsdorf, Germany) and the biostep argusX1 software. The band with the expected size of the DNA sequence with skipped DMD exon 23 (∼280 bp) was cut out, and the DNA was isolated using a QIAquick Gel Extraction Kit (Qiagen, Hilden, Germany). The purified DNA fragment was sequenced (Sanger) by Eurofins GATC Biotech (Konstanz, Germany) with the sequencing primers mCherry-DMDex23_SpliSwi_nested_fwd (5′-GGAGTTCATGCGCTTCAAGG-3′) and mCherry-DMDex23_SpliSwi_nested_rev (5′-GCCGTCCTCGAAGTTCATCA-3′).

### Splicing Modulation *In Vivo*

All animals were handled in accordance with the guidelines of the German Animal Welfare Act and were approved by the animal experiments ethical committee of the “Regierung von Oberbayern”, District Government of Upper Bavaria, Germany.

6-week-old female BALB/c mice (BALB/cByJRj, Janvier, Le Genest-Saint-Isle, France) were housed in isolated ventilated cages under pathogen-free conditions with a 12 h light/dark interval. The mice were acclimated for 7 days prior to treatments. Water and food were provided ad libitum. For splice switching in the physiological *DMD* gene, PMO formulations were prepared 48 h before intravenous injection, as described above. The freeze-dried PMO formulations were reconstituted with HBG to obtain a concentration of 375 μg PMO per 150 μL. The mice were randomly divided into three groups (*n* = 6 for PMO(Ex23)-1395, and *n* = 5 for free 3′ primary amine-modified PMO(Ex23) and PMO(705)-1395). 150 μL of PMO solution was injected intravenously into a lateral tail vein. 48 h after injection, the mice were euthanized and the brain, spleen, quadriceps femoris muscle, kidneys, liver, lung, and heart were harvested. For stabilization of the mRNA, the organs were incubated in RNAlater solution (Thermo Fisher Scientific, Waltham) overnight at 4 °C and afterward stored at −20 °C. Each organ was manually homogenized using a mortar and pestle and liquid nitrogen. The mRNA was isolated using the peqGOLD Total RNA Kit (VWR International, Darmstadt, Germany). 400 ng of the RNA was used to generate cDNA using the qScript cDNA synthesis kit (Quanta Biosciences, Gaithersburg). To amplify the region of interest, 300 ng of the cDNA was used to perform a PCR with Taq polymerase (New England Biolabs, Ipswich), the primers DMD_Ex20–26 fwd (5′- CAGAATTCTGCCAATTGCTGAG-3′)^54^ and DMD_Ex20–26 rev (5′-TCACCAACTAAAAGTCTGCATTG-3′),^55^ and the following conditions: initial denaturation (94 °C, 30 s), 30 cycles (94 °C, 30 s/55 °C, 1 min/68 °C, 1 min), and final extension (68 °C, 5 min).

The PCR products were purified using a PCR purification kit (Qiagen, Hilden, Germany). 100 ng of the purified PCR product was used to perform a second PCR with the primers DMD_Ex20–24 fwd (5′- CCCAGTCTACCACCCTATCAGAGC-3′)^54^ and DMD_Ex20–24 rev (5′- CAGCCATCCATTTCTGTAAGG-3′),^56^ and the following conditions: initial denaturation (94 °C, 30 s), 30 cycles (94 °C, 30 s/57 °C, 1 min/68 °C, 1 min), and final extension (68 °C, 5 min).

The final PCR products were analyzed by agarose gel electrophoresis (2% agarose gel; 100 V; 2 h). Individual band intensities were quantified and put into relation to the band of full-length DMD-Ex20–24 with a size of 633 bp by using ImageJ software.

To confirm the determined exon 23 skipping in PMO(Ex23)-1395 treated mice, the bands of the lung of animal C1 were purified by gel extraction using a QIAquick Gel Extraction Kit (Qiagen, Hilden, Germany). The purified sequences were sequenced (Sanger) by Eurofins GATC Biotech (Konstanz, Germany) with the primers DMD-Ex20–24 fwd and DMD-Ex20–24 rev at a concentration of 10 ng/μL.

## Notes

Splicing reporter plasmids pEGFP-N1/mCherry-DMDEx23 (#211367) and PB-mCherry-DMDEx23-eGFP (#211366) are deposited at Addgene.
