# Supplementary material for: mCherry on Top: A Positive Read-Out Cellular Platform for Screening DMD Exon Skipping Xenopeptide–PMO Conjugates
Source: Bioconjug Chem. 2023 Nov 22;34(12):2263–74. doi: 10.1021/acs.bioconjchem.3c00408 (PMC10739591; doi:10.1021/acs.bioconjchem.3c00408)
Supplement: Supplementary file 1 — bc3c00408_si_001.pdf [file bc3c00408_si_001.pdf]

Supporting Information

mCherry on Top: A Positive Read-Out Cellular Platform for Screening DMD Exon Skipping Xenopeptide-PMO Conjugates

Anna-Lina Lessl,[a] Jana Pöhmerer,[a] Yi Lin,[a] Ulrich Wilk,[a] Miriam Höhn,[a] Elisa Hörterer,[a] Ernst Wagner,[a,b] Ulrich Lächelt[a,b,c*]

a Pharmaceutical Biotechnology, Department of Pharmacy, LMU Munich, Butenandtstrasse 5-13, 81377 Munich, Germany

b Center for NanoScience (CeNS), LMU Munich, 80799 Munich, Germany

c Department of Pharmaceutical Sciences, University of Vienna, Josef-Holaubek-Platz 2, 1090 Vienna, Austria

* Corresponding Author. Email: ulrich.laechelt@univie.ac.at

Contents

Materials....2

Methods....4

- Synthesis of xenopeptides....4
- PMO functionalization - Synthesis of PMO-DBCO....5
- Generation of PB-CAG-mCherry-DMDEx23-eGFP plasmid....5
- Generation of stable HeLa mCherry-DMDEx23 cells....6
- Splice-switching and luciferase activity assay *in vitro*....6
- Primer list....6
- Statistical analysis....7

Supporting Figures and Tables....8

References....14

Materials

Table S1. List of chemicals and solvents used for the research.

Chemicals and solvents (abbreviations)	Manufacturer
1-Hydroxybenzotriazole (HOBt)	Sigma-Aldrich, Munich, Germany
2-(1H-benzotriazol-1-yl)-1,1,3,3-tetramethyluronium Hexafluorophosphate (HBTU)	Multisyntech, Witten, Germany
2-Chlorotritylchloride resin	Iris Biotech, Marktredwitz, Germany
4′,6-diamidino-2-phenylindole (DAPI)	Sigma-Aldrich, Munich, Germany
Acetonitrile (ACN)	VWR Int., Darmstadt, Germany
Adenosine triphosphate (ATP)	Roche, Penzberg, Germany
Agarose	Sigma-Aldrich, Munich, Germany
Ampicillin	Sigma-Aldrich, Munich, Germany
Benzotriazol-1-yl-oxy tripyrrolidinophosphonium hexafluorophosphate (Pybop®)	Multisyntech, Witten, Germany
Cell culture lysis buffer	Promega, Mannheim, Germany
Coenzyme A	Sigma-Aldrich, Munich, Germany
Collagen	Biochrom, Germany
Dibenzocyclooctyne-*N*-hydroxysuccinimidyl ester (DBCO-NHS ester)	Sigma-Aldrich, Munich, Germany
Dichloromethane (DCM)	Bernd Kraft, Duisburg, Germany
Dimethyl sulfoxide (water free) (DMSO)	Sigma-Aldrich, Munich, Germany
Di-*tert*-butyl dicarbonate (Boc anhydride)	Sigma-Aldrich, Munich, Germany
D-luciferin sodium	Promega, Mannheim, Germany
Dulbecco’s Modified Eagle’s Medium (DMEM)	Life Technologies (Carlsbad, USA).
Ethanol absolute (EtOH)	VWR Int., Darmstadt, Germany
Ethylenediaminetetraacetic acid disodium salt dihydrate (EDTA)	Sigma-Aldrich, Munich, Germany
Fatty acids	Sigma-Aldrich, Munich, Germany
Fetal bovine serum (FBS)	Life Technologies, Carlsbad, USA
Fmoc-α-amino acids	Iris Biotech, Marktredwitz, Germany
Fmoc-Stp(Boc3)-OH building block	In-house synthesis [1]
GelRed	Sigma-Aldrich, Munich, Germany
Glycylglycine	Sigma-Aldrich, Munich, Germany
HEPES (*N*-(2-hydroxyethyl)piperazine-N´-(2-ethanesulfonic acid)	Biomol GmbH, Hamburg, Germany
Hydrazine monohydrate	Sigma-Aldrich, Munich, Germany
Hydrochloric acid (HCl) solution (1 M)	Bernd Kraft, Duisburg, Germany
Isopropanol	Merck, Darmstadt, Germany
Lipofectamine 3000	ThermoFisher Scientific, Schwerte, Germany

Methyl *tert*-butyl ether (MTBE)	Brenntag GmbH, Essen, Germany
MTT (3-(4,5-dimethylthiazol-2-yl)-2,5-diphenyltetrazolium bromide)	Sigma-Aldrich, Munich, Germany
N,N-Diisopropylethylamine (DIPEA)	Iris Biotech, Marktredwitz, Germany
N,N-Dimethylformamide (DMF)	Iris Biotech, Marktredwitz, Germany
n-hexane	Grüssing GmbH, Filsum, Germany
N-Methyl-2-pyrrolidone (NMP)	Iris Biotech, Marktredwitz, Germany
Nuclease-free water	Sigma-Aldrich, Munich, Germany
Paraformaldehyde	Sigma-Aldrich, Munich, Germany
Penicillin/Streptomycin	Life Technologies (Carlsbad, USA).
peqGold 1KB DNA ladder	VWR Int., Darmstadt, Germany
Piperidine	Iris Biotech, Marktredwitz, Germany
Rhodamine phalloidin	Life Technologies, Carlsbad, USA
Sodium Acetate	Sigma-Aldrich, Munich, Germany
Trifluoro acetic acid (TFA)	Iris Biotech, Marktredwitz, Germany
Triisopropylsilane (TIS)	Sigma-Aldrich, Munich, Germany
Trypsin/EDTA	PANBiotech, Aidenbach, Germany

Table S2. List of PMOs used for the research.

PMO	Target	PMO Sequence	Supplier
PMO(705)	G point mutation at position 705 in intron 2 of the human β-globin gene (IVS2-705)	CCTCTTACCTCAGTTACAATTTATA*	Gene Tools, LLC, Philomath, OR, USA
PMO(Ex23)	Donor splice site of DMD intro 23	GGCCAAACCTCGGCTTACCTGAAAT*	Gene Tools, LLC, Philomath, OR, USA

*PMOs contained a 3'-primary amine (PMO-NH_2) for DBCO functionalization.

Deionized water was purified in-house using an Evoqua Ultra Clear® Glass Panel Systems (Günzburg, Germany) and was used for all experiments.

Kaiser test solutions: 80 % (w/v) phenol in EtOH; 5 % (w/v) ninhydrine in EtOH; 20 µM KCN in pyridine (2 mL of 1 mM potassium cyanide (aq) in 98 mL of pyridine).[2]

HEPES buffered glucose (HBG) buffer was prepared by dissolving 2.38 g HEPES (10 mmol) and 27.5 g glucose monohydrate in 490 mL water (20 mM HEPES, 5 % w/v glucose, pH 7.4). The pH was adjusted to 7.4 by the addition of NaOH and water was added to a final volume of 500 mL.

For 10x NET gelatine, 25 g gelatina alba was dissolved in 1000 mL 10x NET (1.5 M NaCl, 0.05 % EDTA (pH 8.0), 0.5 M Tris (pH 7.5), 0.5 % Triton-X-100) while boiling. For usage, the 10x NET gelatine was diluted with demineralized water 1:10 to 1x NET gelatine.

Methods

Table S3. List of xenopeptides used for the research.

xenopeptide ID	Sequence (N→C)	Reference
1195	K(N_3)-Y_3-Stp_2-K[G-K(LenA)$_2$]-Stp_2-Y_3	[3]
1391	K(N_3)-Y_3-Stp-K(K(OleA)$_2$)-Stp-Y_3	[4]
1392	K(N_3)-Y_3-Stp-K(K(LinA)$_2$)-Stp-Y_3	[4]
1393	K(N_3)-Y_3-Stp-K(K(LenA)$_2$)-Stp-Y_3	[4]
1395	K(N_3)-Y_3-H-Stp-H-K(K(OleA)$_2$)-H-Stp-H-Y_3	[4]
1396	K(N_3)-Y_3-H-Stp-H-K(K(LinA)$_2$)-H-Stp-H-Y_3	[4]
1397	K(N_3)-Y_3-H-Stp-H-K(K(LenA)$_2$)-H-Stp-H-Y_3	[4]

Synthesis of xenopeptides

The azide-containing xenopeptides used for conjugation and formulation of PMOs were synthesized and characterized as described previously with minor modification [3, 4]. Table S3 provides an overview over the individual sequences, internal ID numbers and further descriptions. Briefly, sequence-defined lipo-xenopeptides (XP) were synthesized by standard Fmoc solid-phase peptide synthesis. A 2-chlorotrityl chloride resin was used, which was preloaded with the first amino acid Tyr(tBu)-OH. The artificial Fmoc- and Boc-protected amino acid Fmoc-Stp(Boc)$_3$-OH was synthesized as previously reported [1, 5]. The sequences of the α-peptide backbones, excluding the N-terminal K(N_3) and side-chain lipid modification, were synthesized from C-terminus to N-terminus using a SyroWave synthesizer (Biotage, Uppsala, Sweden). Every coupling step was carried out twice with 4 eq. Fmoc-amino acid, 4 eq. HOBt, 4 eq. HBTU, and 8 eq. DIPEA in NMP/DMF (5 mL/g resin) for 12 minutes at 50 °C. Fmoc deprotection was carried out by 4 times incubation with 20 % piperidine in DMF (7 mL/g resin) for 10 minutes. After each coupling and deprotection step, the resin was washed 5 times with DMF (10 mL/g resin) for 1 minute each. By including Fmoc-L-Lys(Dde)-OH in the backbone, an asymmetric branching point was introduced.

The N-terminal Fmoc-L-Lys(N_3)-OH coupling as well side-chain modifications of K(Dde) were performed manually as follows. Manual couplings were conducted at RT with 4 eq. Fmoc-protected amino acid, 4 eq. HOBt, 4 eq. PyBOP, and 8 eq. DIPEA in DCM/DMF (50/50) (10 mL/g resin) for 90 minutes using syringe microreactors and an overhead shaker followed by washing the resin three times with DMF and three times with DCM (10 mL/g resin each). The Fmoc protecting group was removed by incubating the resin with 20 % piperidine in DMF (10 mL/g resin) four times for ten minutes. After the coupling and deprotection steps, a Kaiser test [2] was performed to confirm the accomplishment of the previous step. The N-terminus was protected with a Boc group by using 10 eq. Boc anhydride and 10 eq. DIPEA in DCM/DMF (10 mL/g resin). To enable chain elongation at the ε-amine of the Dde-protected lysine, the resin was incubated with 2 % hydrazine hydroxide (v/v) in DMF (10 mL/g resin) for three times. The first and third incubation was performed for 5 minutes, the second incubation step took 7 minutes. The resin was washed for 10 times with DMF (10 mL/g resin), five times with 10 % DIPEA (V/V) in DMF and five times with DCM. Fmoc-Lys(Fmoc)-OH was coupled and deprotected manually as described above. The final coupling step was performed by incubating the resin with 8 eq. of the desired fatty acid, 8 eq. HOBt, 8 eq. PyBOP, and 16 eq. DIPEA in DCM/DMF (50/50) (10 mL/g resin) for 90 minutes. After drying of the resin, the peptides were cleaved by using a cocktail consisting of trifluoroacetic acid (TFA)/triisopropylsilane (TIS)/H_2O (95/2.5/2.5 v/v/v) (10 mL/g resin). The cleavage cocktail was cooled to 4 °C to avoid TFA-adduct formation,[6] added to the resin and incubated at RT for 20 minutes. Afterwards, the cleavage solution was transferred rapidly into 40 mL of pre-cooled methyl-*tert*-butylether (MTBE)/*n*-hexane (50/50 v/v). The mixture was centrifuged, the supernatant was discarded and precipitated peptide dried. Purification of the peptide was conducted by size exclusion chromatography

using an Äkta purifier system (GE Healthcare Bio-Sciences AB, Sweden) equipped with a P-900 solvent pump module, a UV-900 spectrophotometric detector, a pH/C-900 conductivity module, a Frac-950 automated fractionator, a Sephadex G-10 column and 10 mM HCl in H_2O/ACN (70/30 v/v) as solvent. Product fractions were combined and lyophilized to obtain the final product.

PMO functionalization - Synthesis of PMO-DBCO

1 µmol of 3' primary amine modified PMO (Gene Tools, USA) was dissolved in 300 µL water-free DMSO. 2.5 mg of DBCO-NHS ester (Sigma-Aldrich, Germany) was dissolved in 100 µL water-free DMSO and 0.3 µL DIPEA was added. The solutions were combined and incubated overnight at RT under shaking at 300 rpm.

The DBCO functionalized PMO was purified by size exclusion chromatography (SEC) using the Äkta purifier system (GE Healthcare Bio-Sciences AB, Uppsala, Sweden) comprising a P-900 solvent pump module, a UV-900 spectrophotometric detector, a pH/C-900 conductivity module, a Frac-950 automated fractionator, and a Sephadex G-10 column. The solvent composition was 30 % ACN in Millipore water. The pooled product containing fractions were lyophilized, dissolved in water and analyzed by MALDI-MS. The PMO-DBCO solution concentration was determined photometrically at λ = 265 nm using the extinction coefficient provided by the PMO supplier.

Transmission electron microscopy (TEM)

PMO-XP formulations were prepared in HEPES as described in the main manuscript. Samples contained either PMO(Ex23)-DBCO alone or PMO(Ex23)-XP formulations at a molar ratio of 1:3 PMO to XP. Carbon coated copper grids (Ted Pella, Inc. USA, 300 mesh, 3.0 mm O. D.) were hydrophilized with a plasma cleaner under argon atmosphere (420 V, 1 min). The grids were placed with the activated face down on top of 10 µL sample droplets for 20 s. Afterwards, the sample was removed with a filter paper and stained using a two-step process: first, the grid was washed with 5 µL staining solution (1.0 % uranyl formate in water), which was removed immediately. Second, 5 µL of the same solution was left on the grid for 5 s. Afterwards, it was removed with a filter paper and grids were allowed to dry for 20 min. Grids were stored at room temperature. The samples were measured on a JEOL JEM-1100 electron microscope using 80 kV acceleration voltage.

Generation of PB-CAG-mCherry-DMDEx23-eGFP plasmid

For the generation of stable mCherry-DMDEx23 reporter cells, the reporter gene construct was subcloned from pEGFP-N1/mCherry-DMDEx23 obtained from BioCat GmbH (Heidelberg, Germany) into a PiggyBac plasmid. PB-CAG-GFPd2 was a gift from Jordan Green (AddGene plasmid #115665; http://n2t.net/addgene:115665; RRID:Addgene_115665). First, the *GFPd2* gene was cleaved out of PB-CAG-GFPd2 using the restriction enzymes XmaI (New England Biolabs, Ipswich, USA) and NotI (New England Biolabs, Ipswich, USA) in sequence. Intermediate and final purification was performed by gel electrophoresis (1 % agarose; 100 V; 3 h) followed by gel extraction. The *mCherry-DMDEx23-eGFP* insert was generated by PCR amplification from pEGFP-N1/mCherry-DMDEx23 using Taq DNA polymerase (NEB) and PCR primers which include NotI and XmaI restriction sites at the 5' ends. The primers mCherry-DMDex23-eGFP_fwd (5'-ATCCCGGGGACTCAGATCTCGAGGCCACCATG-3'), mCherry-DMDex23-eGFP_rev (5'-TCGCGGCCGCTTTACTTGTACAGCTC-3') and following PCR conditions were used: initial denaturation (94 °C, 30 sec), 30 cycles (94 °C, 30 sec / 60 °C, 1 min / 68 °C, 1 min), final extension (68 °C, 5 min). The *mCherry-DMDEx23-eGFP* PCR product was purified by electrophoresis on a 1 % agarose gel. Sticky ends were generated by digestion of the PCR amplicon with XmaI and NotI. Intermediate and final purification was performed by gel electrophoresis (1 % agarose; 100 V; 3 h) followed by gel extraction. The obtained mCherry-DMDEx23-eGFP insert (~1291 bp) was integrated into the PB-CAG vector (5592 bp) by using standard cloning techniques. DH5alpha cells were transformed with the ligation reaction mixture and PB-CAG-mCherry-DMDEx23-eGFP plasmid was isolated using Qiagen Plasmid Maxi kit.

Generation of stable HeLa mCherry-DMDEx23 cells

Hela wt cells were seeded in a 48-well plate at a density of 12,500 cells/well 24 h before transfection. The next day, 500 ng Super piggyBac Transposase expression vector (SBI, CA, USA) and 200 ng PB-CAG-mCherry-DMDEx23-eGFP plasmid were co-transfected using Lipofectamine3000 (ThermoFisher Scientific, USA) according to the manufacturer's protocol. 48 h after pDNA treatment, the transfection mixture containing medium in each well was replaced by fresh medium. 3 days, 17 days and 22 days after treatment, the cells were evaluated by flow cytometry as described in the main manuscript. The fluorescence of cells was determined by excitation of DAPI at 405 nm, detection of emission at 450 nm, excitation of eGFP at 488 nm and detection of emission at 530 nm. Only isolated and viable cells were evaluated. Flow cytometry data was analyzed using FlowJo X 10.0.7r2 flow cytometric analysis software by FlowJo, LLC (Becton, Dickinson and Company, USA). After expansion, cells were sorted to isolate viable and eGFP expressing cell populations with a BD FACSAriaFusion. The cell sorting was performed at the Core Facility Flow Cytometry of the Biomedical Center, LMU Munich. Flow cytometry data was analyzed using FlowJo (Tree Star Inc.). Single cell clones were generated from the sorted polyclonal population of HeLa mCherry-DMDEx23 cells using limiting dilution method in 96-well plates. A suitable monoclonal HeLa mCherry-DMDEx23 cell line was selected by determining eGFP and mCherry expression after PMO(Ex23) treatments.

Splice-switching and luciferase activity assay *in vitro*

24 h prior to transfection, 96-well plates (Corning ® Costar, Sigma-Aldrich, Germany) were coated with collagen and HeLa pLuc/705 cells were seeded at a density of 5,000 cells/well. 50 µM PMO-XP formulation was prepared as explained in the main manuscript. Prior to transfection, the medium in the 96-well plates was replaced by 90 µL fresh medium, and 10 µL of the PMO(705)-XP formulation was added into each well. Cells were incubated at 37 °C and 5 % CO_2 in a humidified atmosphere for 24 h. After incubation, the medium was removed and 100 µL of 0.5x cell lysis buffer (25 mM Tris, pH 7.8; 2 mM EDTA; 1 mM DTT; 10 % glycerol, 1 % Triton X-100) was added to each well to lyse the cells. The well plates were incubated for 30 minutes at RT. After the incubation, the 96-well plates were stored at -80 °C. The luminescence measurements were performed by using a Centro LB 960 plate reader luminometer from Berthold Technologies (Bad Wildbad, Germany). 500 µL of a 10 mM luciferin solution was diluted with 9.5 mL LAR buffer (20 mM glycylglycine; 1 mM MgCl2; 0.1 mM EDTA; 3.3 mM DTT; 0.55 mM adenosine 5′-triphosphate; 0.27 mM coenzyme A, pH 8-8.5). 35 µL of each cell lysate was transferred to a luminometer microplate and 100 µL luciferin-LAR buffer was added to each well before the measurement. With a delay of 2 seconds, the relative light units were measured for 10 seconds. To relate the luminescence of the treated cells to the background of untreated cells, the mean value of each group was divided by the mean of the HBG treated negative control cells and a 'fold increase' value was calculated for each group.

Table S4. List of PCR primers used for the research.

Primer name	Sequence
mCherry-DMDex23-eGFP_fwd	5'-ATCCCGGGGACTCAGATCTCGAGGCCACCATG-3'
mCherry-DMDex23-eGFP_rev	5'-TCGCGGCCGCTTTACTTGTACAGCTC-3'
mCherry-DMDex23_SpliSwi_fwd	5'-GGAGGATAACATGGCCATCA-3'
mCherry-DMDex23_SpliSwi_rev	5'-GTCCTTCAGCTTCAGCCTCT-3'
mCherry-DMDex23_SpliSwi_nested_fwd	5'-GGAGTTCATGCGCTTCAAGG-3'
mCherry-DMDex23_SpliSwi_nested_rev	5'-GCCGTCCTCGAAGTTCATCA-3'
DMD_Ex20-26 fwd	5'-CAGAATTCTGCCAATTGCTGAG-3' [7]
DMD_Ex20-26 rev	5'-TCACCAACTAAAAGTCTGCATTG-3' [8]
DMD_Ex20-24 fwd	5'-CCCAGTCTACCACCCTATCAGAGC-3' [7]
DMD_Ex20-24 rev	5'-CAGCCATCCATTTCTGTAAGG-3' [9]

Statistical analysis

Data was analyzed using GraphPad prism 6. The statistical significance of the experiments was estimated using the two-tailed student's t-test, **** $p \leq 0.0001$, *** $p \leq 0.001$, ** $p \leq 0.01$, * $p \leq 0.05$. ns $p > 0.05$. Data are presented as mean ± SD.

Supporting Figures and Tables

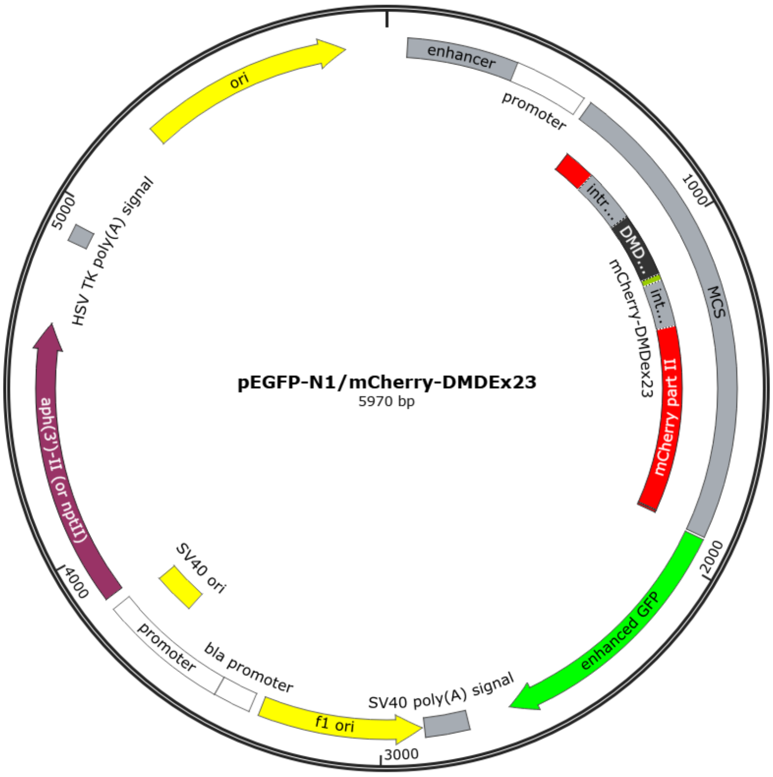

Figure S1 Plasmid map of pEFGP-N1/mCherry-DMDEx23.

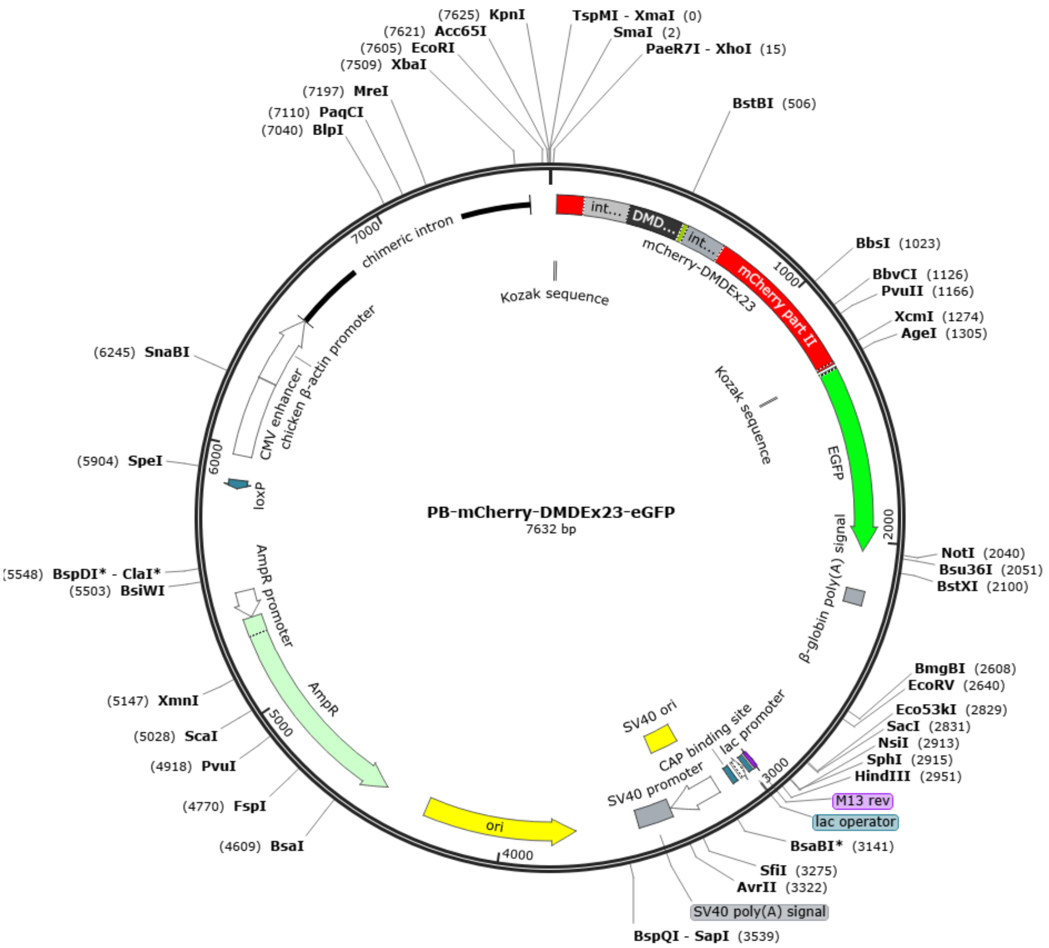

Figure S2 Plasmid map of PB-mCherry-DMDEx23-eGFP.

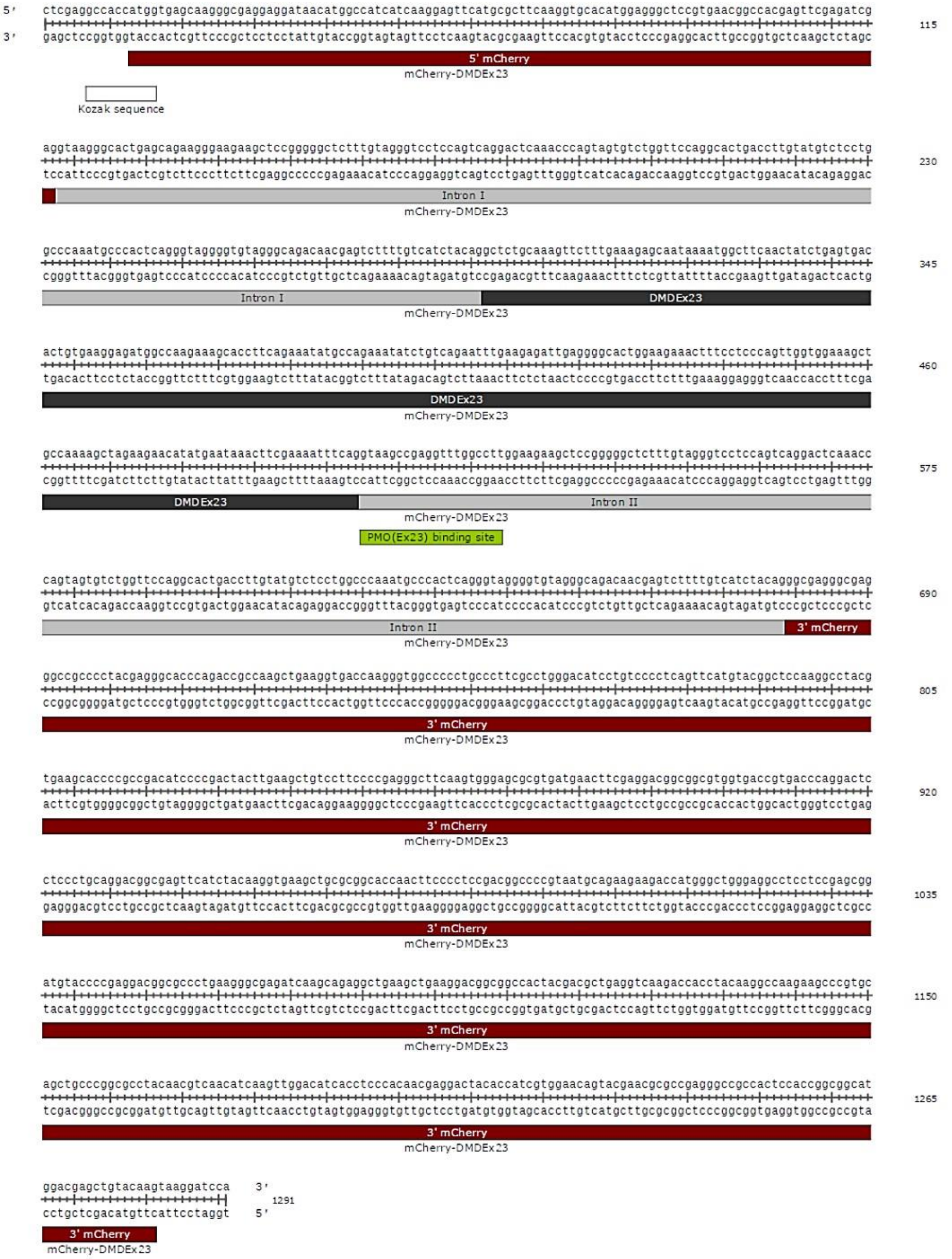

Figure S3 Sequence of the reporter gene mCherry-DMDEx23 with separate section labels and PMO(Ex23) binding site.

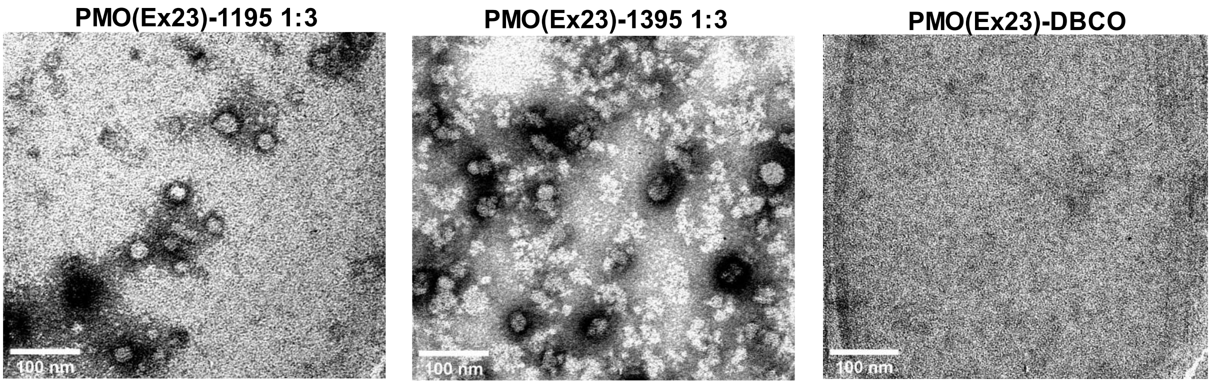

Figure S4 Transmission electron microscopy (TEM) images of unformulated PMO(Ex23)-DBCO or formulations with #1195 and #1395 at a molar ratio of 1:3 (PMO:XP).

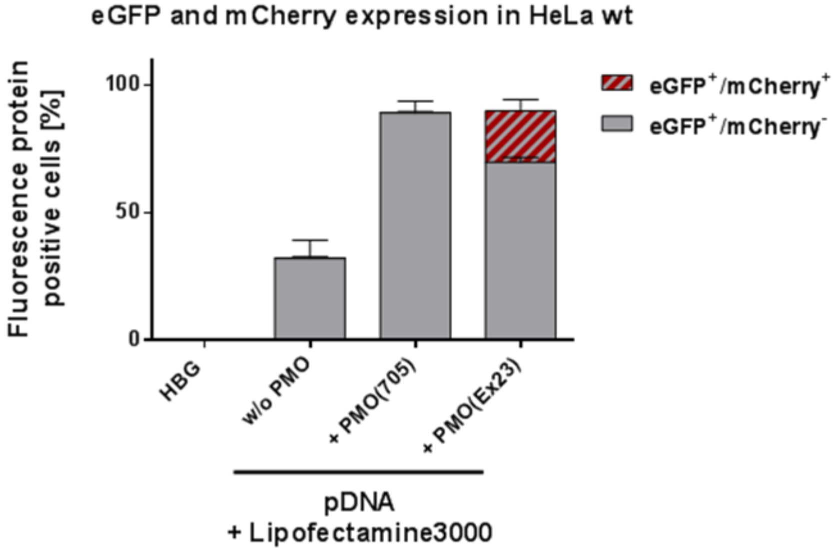

Figure S5 eGFP and mCherry expression of HeLa wt cells after transient transfection with pEGFP-N1/mCherry-DMDEx23 and subsequent treatment with PMO-1195 formulations. 200 ng pEGFP-N1/mCherry-DMDEx23 per well was transfected with Lipofectamine 3000. PMO(705)-DBCO and PMO(Ex23)-DBCO were conjugated and formulated with XP #1195 at a molar ratio of 1:3 (PMO:XP) and used at a concentration of 2.5 µM.

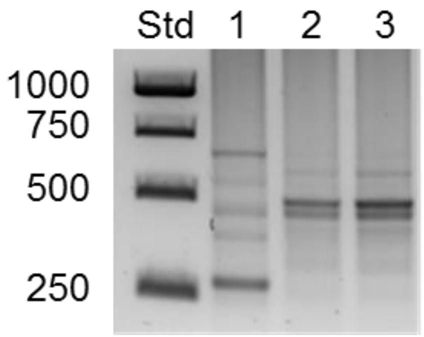

Figure S6 Detection of mdx exon 23 skipping of mCherry-DMDEx23 mRNA by RT-PCR. Total RNA was extracted from cells 24h after PMO(Ex23)-1195 and PMO(705)-1195 treatment (2.5 µM PMO). A sequence surrounding mdx exon23 in mCherry-DMDEx23 was amplified by RT-PCR. The band resulting from mdx exon 23 skipping has a sized of approx. 280 bp. Std: DNA ladder; 1: PMO(Ex23)-1195; 2: PMO(705)-1195; 3: HBG.

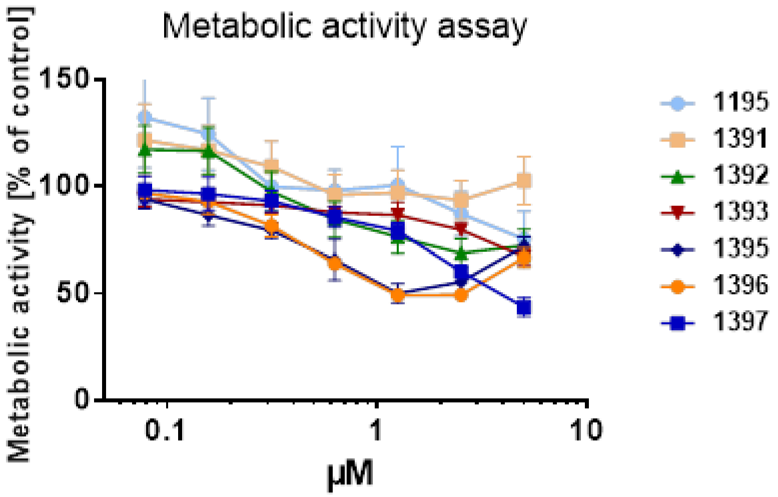

Figure S7 Dose-dependent effects of PMO(Ex23)-xenopeptide (XP) 1:3 formulations on metabolic acitivity of HeLa mCherry-DMDEx23 cells after 24 h treatment. PMO treatments ranged from 0.078125 to 5 µM.

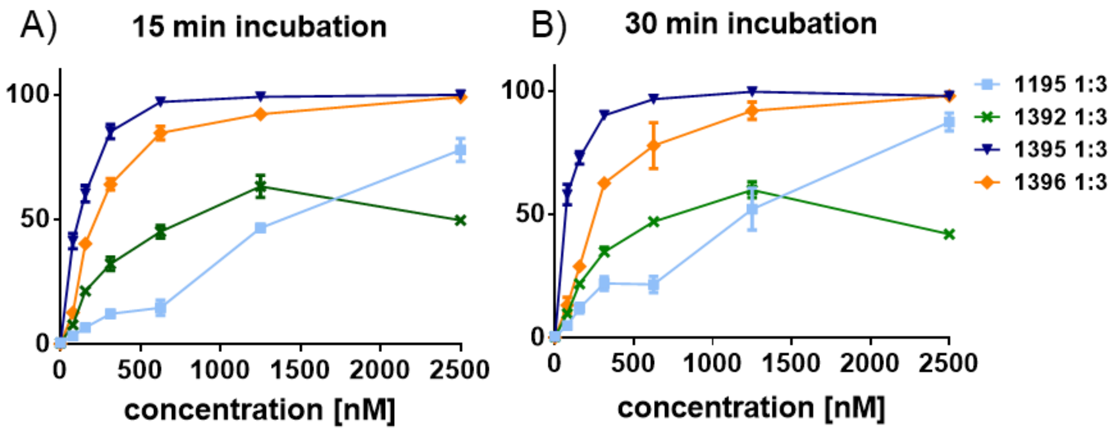

Figure S8 mCherry expression of HeLa mCherry-DMDEx23 cells after 15 min (**A**) and 30 min (**B**) exposure to different PMO(Ex23)-XP formulations and subsequent incubation in fresh medium until 24 h. Data are presented as mean ± SD (n = 3).

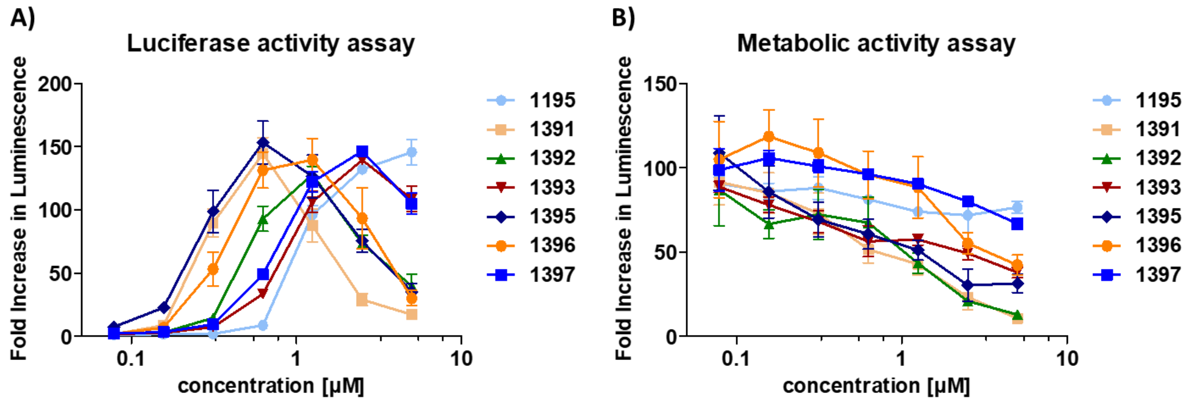

Figure S9 Splice-switching activity of PMO(705)-XP formulations in HeLa pLuc/705 cells. A) Fold increase in luminescence and **B)** metabolic activity 24 h after treatment with PMO-XP formulations (0.078 to 5.0 µM PMO). Data are presented as mean ± SD (n = 3).

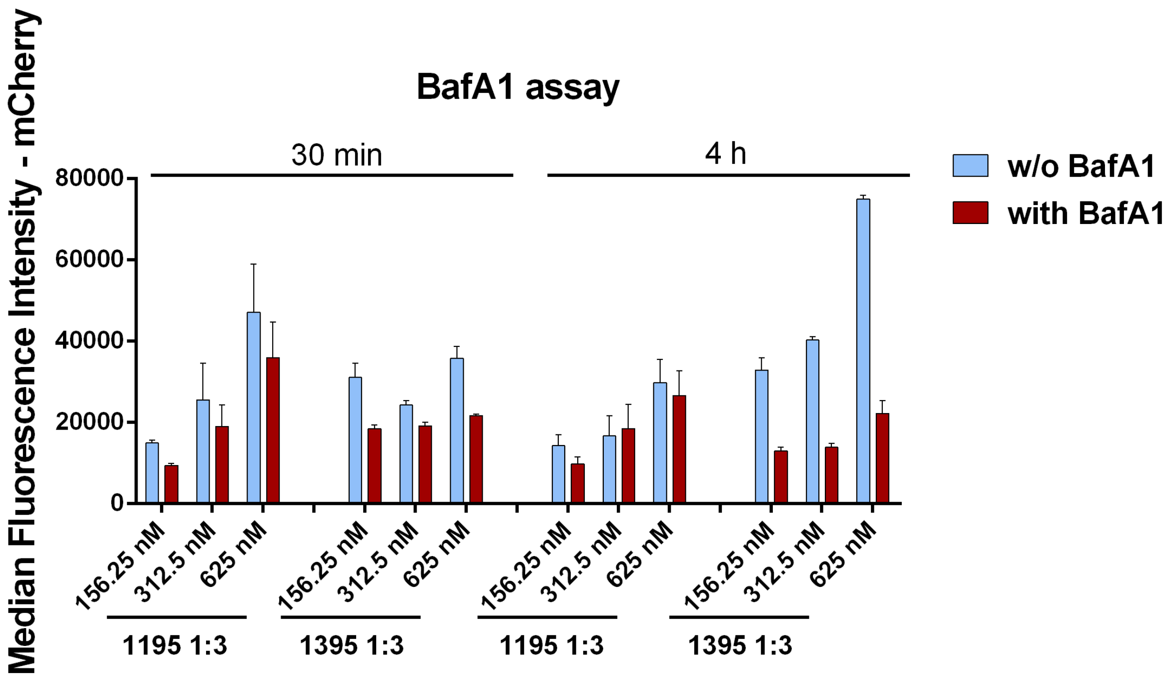

Figure S10 Influence of endosomal acidification on median fluorescence intensitiy (MFI) of mCherry positive cells after PMO-XP treatment. HeLa mCherry-DMDEx23 were pre-incubated with the V-ATPase inhibitor bafilomycin A1 (BafA1, 200 nM) for 2 h and cells were treated with PMO(Ex23)-1195 or -1395 formulations. Medium was replaced by fresh medium 30 min or 4 h after treatment and mCherry positive cells were quantified after a total incubation time of 24 h.

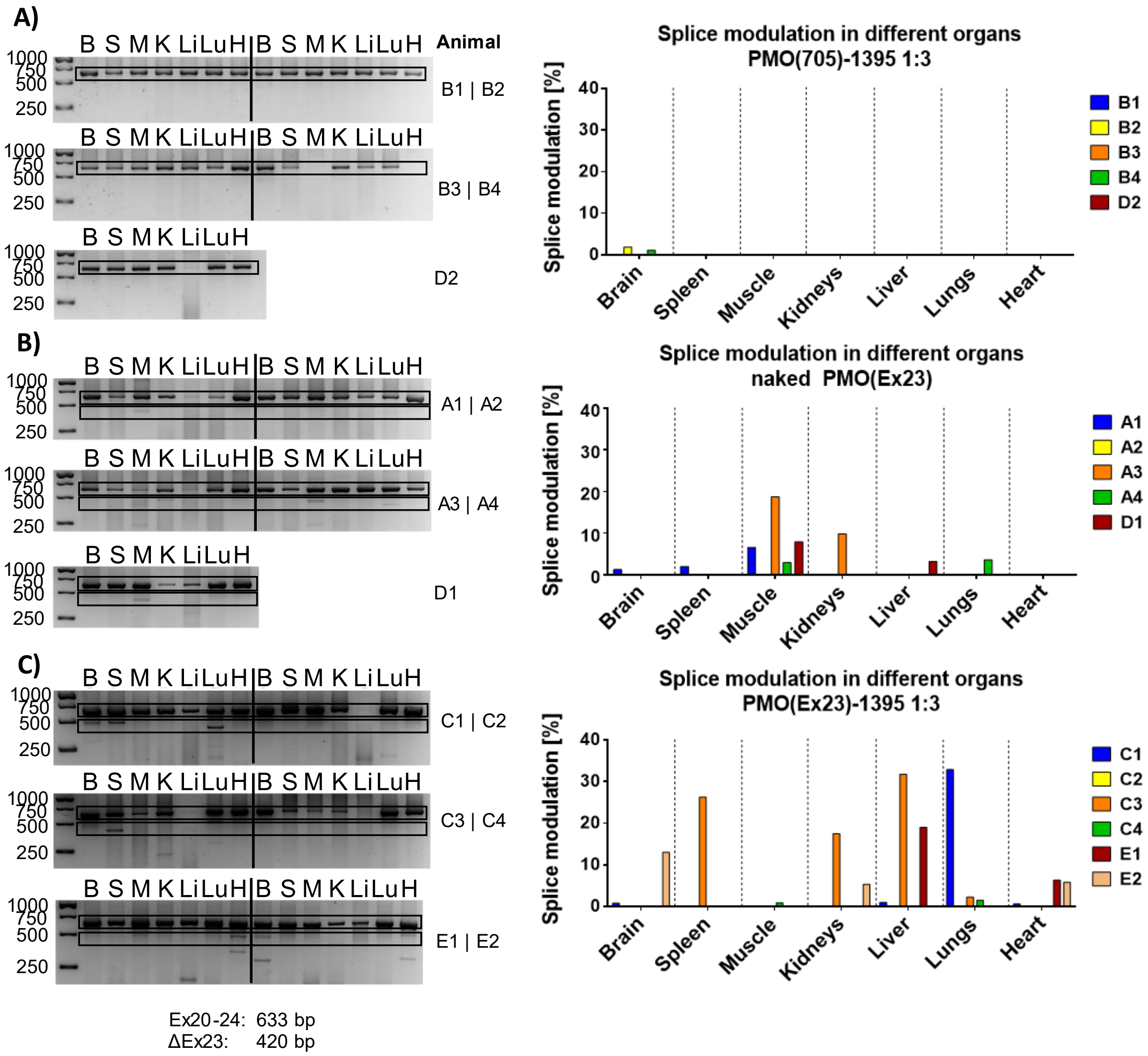

Figure S11 ***In vivo*** **splicing modulation of DMD mRNA in BALB/c mice determined by RT-PCR.** Total RNA was extracted from homogenized organs 48 h after intravenous injection of PMO formulations (375 µg PMO) and nested RT-PCR was conducted to amplify DMD Ex20-24. **A)** PMO(705)-1395 1:3 (n=5), **B)** naked unmodified PMO(Ex23) (n=5), and **C)** PMO(Ex23)-1395 1:3 (n=6). Single organs are indicated as: "B" = brain, "S" = spleen, "M" = quadriceps femoris muscle, "K" = kidneys, "Li" = liver, "Lu" = lung, "H" = heart. Ratios of splicing modulation were determined using ImageJ Software and are shown on the right side of each data set.

References

1. Schaffert, D., N. Badgujar, and E. Wagner, *Novel Fmoc-Polyamino Acids for Solid-Phase Synthesis of Defined Polyamidoamines.* Organic Letters, 2011. **13**(7): p. 1586-1589.
2. Kaiser, E., et al., *Color test for detection of free terminal amino groups in the solid-phase synthesis of peptides.* Analytical Biochemistry, 1970. **34**(2): p. 595-598.
3. Kuhn, J., et al., *Supramolecular Assembly of Aminoethylene-Lipopeptide PMO Conjugates into RNA Splice-Switching Nanomicelles.* Advanced Functional Materials, 2019. **29**(48).
4. Lin, Y., et al., *Chemical Evolution of Amphiphilic Xenopeptides for Potentiated Cas9 Ribonucleoprotein Delivery.* J Am Chem Soc, 2023. **145**(28): p. 15171-15179.
5. Schaffert, D., et al., *Solid-phase synthesis of sequence-defined T-, i-, and U-shape polymers for pDNA and siRNA delivery.* Angew Chem Int Ed Engl, 2011. **50**(38): p. 8986-9.
6. Reinhard, S., W. Zhang, and E. Wagner, *Optimized Solid-Phase-Assisted Synthesis of Oleic Acid Containing siRNA Nanocarriers.* ChemMedChem, 2017. **12**(17): p. 1464-1470.
7. Hammond, S.M., et al., *Correlating In Vitro Splice Switching Activity With Systemic In Vivo Delivery Using Novel ZEN-modified Oligonucleotides.* Mol Ther Nucleic Acids, 2014. **3**(11): p. e212.
8. Gee, P., et al., *Extracellular nanovesicles for packaging of CRISPR-Cas9 protein and sgRNA to induce therapeutic exon skipping.* Nat Commun, 2020. **11**(1): p. 1334.
9. Yin, H., et al., *Cell-penetrating peptide-conjugated antisense oligonucleotides restore systemic muscle and cardiac dystrophin expression and function.* Hum Mol Genet, 2008. **17**(24): p. 3909-18.
